# Chronic Low-Grade Inflammation and Brain Structure in the Middle-Aged and Elderly Adults

**DOI:** 10.3390/nu16142313

**Published:** 2024-07-18

**Authors:** Yujia Bao, Xixi Chen, Yongxuan Li, Shenghao Yuan, Lefei Han, Xiaobei Deng, Jinjun Ran

**Affiliations:** 1 School of Public Health, Shanghai Jiao Tong University School of Medicine, Shanghai 200025, China; bubble-y@sjtu.edu.cn (Y.B.); c.cctx@sjtu.edu.cn (X.C.); melody321@sjtu.edu.cn (Y.L.); scp-173@sjtu.edu.cn (S.Y.); 2 School of Global Health, Shanghai Jiao Tong University School of Medicine, Shanghai 200025, China; lfhan@sjtu.edu.cn; 3 School of Public Health, University of Hong Kong, Hong Kong SAR, China

**Keywords:** low-grade inflammation, brain structure, epidemiology, urban health

## Abstract

Low-grade inflammation (LGI) mainly acted as the mediator of the association of obesity and inflammatory diet with numerous chronic diseases, including neuropsychiatric diseases. However, the evidence about the effect of LGI on brain structure is limited but important, especially in the context of accelerating aging. This study was then designed to close the gap, and we leveraged a total of 37,699 participants from the UK Biobank and utilized inflammation score (INFLA-score) to measure LGI. We built the longitudinal relationships of INFLA-score with brain imaging phenotypes using multiple linear regression models. We further analyzed the interactive effects of specific covariates. The results showed high level inflammation reduced the volumes of the subcortex and cortex, especially the globus pallidus (β [95% confidence interval] = −0.062 [−0.083, −0.041]), thalamus (−0.053 [−0.073, −0.033]), insula (−0.052 [−0.072, −0.032]), superior temporal gyrus (−0.049 [−0.069, −0.028]), lateral orbitofrontal cortex (−0.047 [−0.068, −0.027]), and others. Most significant effects were observed among urban residents. Furthermore, males and individuals with physical frailty were susceptive to the associations. The study provided potential insights into pathological changes during disease progression and might aid in the development of preventive and control targets in an age-friendly city to promote great health and well-being for sustainable development goals.

## 1. Introduction

Fueled by nutrition transition, a sedentary lifestyle, and modern amenities, the risk for chronic diseases, many of which are inflammatory in origin, increased dramatically [1,2]. Inflammatory diet and obesity in humans were shown to be related to high-level inflammation and might be viewed as the risk factors of chronic diseases such as cardiovascular and neuropsychiatric diseases [3,4,5]. Acute inflammation is the natural process of the body for healing wounds and promoting tissue regeneration in a competent immune response to injury or infection [6]. Low-grade systemic inflammation, on the contrary, was associated with several chronic conditions [7,8,9]. Moreover, it was not only regarded as one of the principal mechanisms underlying the pathophysiology of the above chronic diseases, but also considered to play an important part in treatment-resistant forms of these diseases [10]. The low-grade inflammatory response triggered by diet and obesity involved numerous biomarkers, including circulating (i.e., C-reactive protein [CRP]) and cellular (i.e., leukocyte count) which were found to be relevant to increased risks of chronic diseases as well [11,12,13]. Therefore, identifying the health effects and pathways of low-grade inflammation (LGI) was of major importance in disease prevention and control.

Recently, a number of studies have observed the changes of brain structure among individuals with cardiovascular and neuropsychiatric diseases [14,15]. It is more probable that the structural changes and dysfunction in brain systems may be indicative of the onset of neuropsychiatric disorders. Meanwhile, it has been reported that inflammation is implicated in the structural brain alternations underlying neuropsychiatric disorders [16]. This is due to the effects of microglia and astrocytic function on synaptic pruning, which affects grey matter volume subsequently [16]. Moreover, the changes in grey matter mainly focused on temporal, orbitofrontal and inferior frontal, and cingulate regions [17,18]. To date, relatively few studies have estimated the effect of LGI as measured by an aggregated inflammation score (INFLA-score) on brain structure comprehensively.

To address the vital gap, we investigated the associations between LGI and brain imaging phenotypes based on the UK Biobank. With detailed data on demographics, biochemical index, and lifestyle, we further assess the interactive effects of these factors on the associations. Our study would highlight the importance of LGI in the change of brain structure, which might have considerable implications for the identification of risks and novel treatments.

## 2. Materials and Methods

### 2.1. Study Population

The United Kingdom Biobank (UK Biobank) is a large prospective cohort study of almost half a million participants aged 37–73 years at recruitment during 2006–2010. The targeted population resided within 40 km of 22 assessment centers situated throughout the UK near roads or transit points across England, Scotland, and Wales. The centers covered a diverse range of settings to show the socioeconomic and ethnic heterogeneity, as well as the urban-rural mix. The study participants were administered touchscreen questionnaires, and physical examinations, and provided biological samples. The UK Biobank has been approved by the North West Multi-Center Research Ethics Committee, and all participants had informed consent forms (UK Biobank application number: 99001). The study protocol of the UK Biobank is available online (www.ukbiobank.ac.uk), which are accessed on 1 June 2024. Individuals who withdrew from the cohort study were not eligible for the subsequent analyses. Among the 502,369 study participants, we excluded individuals without data of exposure assessment, specific covariates, and brain imaging phenotypes. Participants with immune trait-specific outliers (i.e., below the first quartile (Q1) subtracted three times the interquartile range (IQR) or above the third quartile (Q3) plus three times the interquartile range (IQR)) were also excluded from analyses. After the exclusions, our analytic dataset was composed of 37,699 participants. Our study followed the Strengthening the Reporting of Observational Studies in Epidemiology reporting guidelines.

### 2.2. Exposure Assessment

The INFLA-score, consisting of CRP, white blood cell, platelet counts, and neutrophil-to-lymphocyte ratio (NLR), was calculated as a useful tool to measure LGI comprehensively [19]. The INFLA-score and its components were reported to be related to diet, stroke, and mental health in previous studies [20,21,22], which might affect the brain structures as well. The elements of the INFLA-score were well-established markers of systemic inflammation and exerted pro-inflammatory influences jointly on a range of biological processes associated with the immune response [19]. The detailed description of these components and calculation of the score are presented in Supplementary Table S1 and Figure S1. Briefly, the deciles of all biomarkers’ levels were generated; the highest deciles (7 to 10) exhibited an increase in score from 1 to 4, while the lowest deciles (1 to 4) demonstrated a negative scoring pattern from −4 to −1; deciles 5 or 6 received a score of zero. In such a way, the values of four components were aggregated to derive the INFLA-score which represented the extent of LGI, with a range of −16 to 16. A higher INFLA-score indicated more severe LGI. The low-grade INFLA-score was defined and generated in the Moli-sani cohort previously [19] and was widely applied and externally validated to evaluate the possible synergistic effects of inflammation biomarkers in the following studies [20,21,22]. Moreover, the INFLA-score was applied in the UK Biobank cohort study as well, which demonstrated low-grade inflammation, greatly represented by the INFLA-score and played an important mediating role [22]. Individuals with the INFLA score were categorized into levels of low (≤0) and high (>0), with the low level as a reference.

### 2.3. Outcome

The brain imaging phenotypes in the UK Biobank were utilized as outcomes, which were derived from quality-controlled T1-weighted neuroimaging data processed by FreeSurfer (version 6.0). Briefly, the neuroimaging data were scanned by a Siemens Skyra 3T scanner (Siemens Healthineers AG, Erlangen, Germany) using a standard Siemens 32-channel head coil [23]. Detailed information on the imaging protocol is available in an open-source document accessed on 8 June 2024 (https://biobank.ndph.ox.ac.uk/showcase/showcase/docs/brain_mri.pdf). Each volume of cortex and subcortex used in our study was acquired from the image-derived phenotypes (IDPs) provided by the UK Biobank [24]. The Qoala-T method was used to verify the output of FreeSurfer, complemented by manually checking the output close to the threshold. Any output that did not pass the quality control was excluded from the IDPs of FreeSurfer. Subcortical volumes (FIRST) (category 1102) and FreeSurfer desikan white (category 192) corresponding to the volumes of 7 subcortical regions and 33 cortical regions were analyzed in our study. The volumes of brain structures were measured in cubic millimeters (mm^3^). Detailed information on brain imaging phenotypes is presented in Supplementary Table S2.

### 2.4. Covariates

Several potential confounders were considered in our study, in accordance with previous studies, including age, sex, waist-to-hip ratio (WHR), index of multiple deprivation (IMD), and healthy lifestyle (never smoking, never drinking, healthy sleep, healthy diet, and regular physical activity). WHR was calculated as the waist circumference divided by the hip circumference, and the results were then categorized into two groups: poor (≥0.9 for men and ≥0.85 for women) and ideal. The IMD was utilized as the assessment method of socioeconomic deprivation for each resident, which consists of seven domains, such as health, education, and others [25]. Furthermore, physical frailty and metabolic syndromes (MetS) were included as well. Physical frailty was quantified by five criteria: weight loss, fatigue, a lack of exercise, a slow walking speed, and a weak grip [26]. The definition of MetS was based on the uniform criteria that reached three or more of the following: reduction in high-density lipoprotein cholesterol and elevated waist circumference, triglycerides, blood pressure, blood glucose, and CRP [27]. Detailed definitions and calculations of healthy lifestyle, physical frailty, and MetS are given in eMethods.

### 2.5. Statistical Analysis

Among the characteristics and brain structures of the study populations, means (standard deviation [SD]) were utilized to describe the continuous variables, and counts (percentages) were utilized to present the categorical variables. The volumes of the cortex and subcortex were standardized through Z-transformation to maintain the same scale. Multiple linear regression models were performed by controlling several covariates simultaneously to eliminate confounding effects, including age (<65/≥65), sex (male/female), IMD (low/high), WHR (poor/ideal), healthy lifestyle (never smoking, never drinking, regular physical activity, healthy sleep, healthy diet) (yes/no), and existing related diseases (hypertension [HTN], diabetes [DM], and stroke) (yes/no). The associations between four components of INFLA-score and brain structures were also estimated separately. The results were shown as βs and the corresponding 95% confidence intervals (CIs). We further conducted separate false-discovery rate (FDR) corrections for all *p* values of the subcortical cortical regions to reduce type I error. 

Additionally, subgroup analyses were performed by stratifying the age (<65 and ≥65), sex (male and female), WHR (poor and ideal), physical frailty (yes and no), MetS (yes and no), and urban residents (yes and no). Meanwhile, the interactive effects of four components (CRP, white blood cell, platelet counts, and NLR) and demographical factors (age and sex) on brain structure were tested as well. The likelihood ratio test between models with and without the interaction term was conducted to estimate the significance of the interaction effects. To examine the robustness of our results, a series of sensitivity analyses were performed. First, the cohort was restricted to individuals with White European ancestry, as non-White participants only constitute approximately 3% of the dataset. Second, the INFLA-scores were trimmed on minimum and maximum (−16 and 16) to alleviate the influence of the extreme values. Third, considering the effects of neuropsychiatric diseases on brain structure, we control the prevalence of dementia and mental health in analyses additionally. Finally, we further adjusted for telomere length in the model since it represents the degree of aging which could also be potential confounders in the associations. We considered a *p* value of less than 0.05 to be statistically significant. Furthermore, all statistical analyses were conducted with a two-sided approach. The statistical analyses were conducted using the R software, version 4.2.2.

## 3. Results

**Figure 1:**
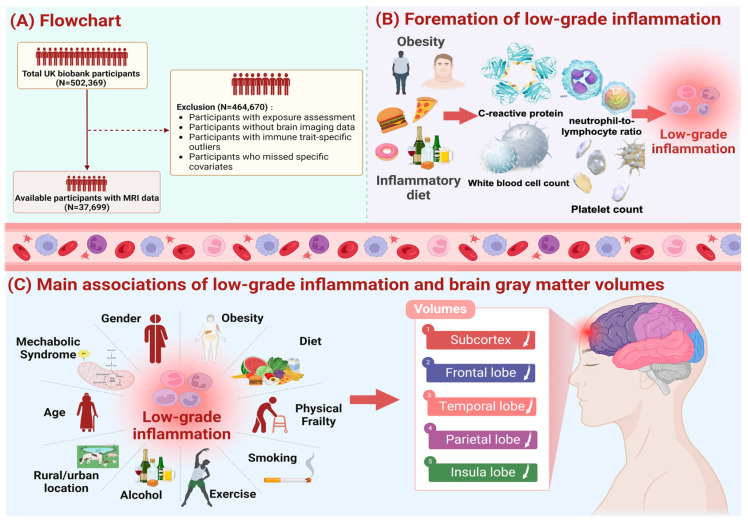
Study workflow. We screened 37,699 UK Biobank participants to explore the effects of low-grade inflammation (LGI) on thd brain system, and the exclusion criteria of our study population is shown in pane (**A**). Additionally, we used the INFLA-score, characterized by C-reactive protein, white blood cell, platelet counts, and neutrophil-to-lymphocyte ratio, to measure and quantify the levels of LGI, and relevant information as shown in pane (**B**). As shown in pane (**C**), taking influential factors of LGI into account, we fit the multiple linear regression model controlling for covariates (age, sex, IMD, WHR, healthy lifestyle, prevalence of hypertension, diabetes mellitus and stroke) and conducted subgroup analysis by age, sex, WHR, metabolic syndrome, physical frailty. The main results demonstrated a significant association of LGI with atrophy of brain regions, including subcortex, frontal lobe, temporal lobe, parietal lobe and insula lobe.

Of the 502,369 UK Biobank individuals, we excluded those without data of exposure, outcome, and specific covariates, leaving 37,699 participants (Figure 1). Supplementary Table S3 presents the details of baseline characteristics. Among the included people, 89.6% were aged less than 65, 53.1% were female, 49.9% had low socioeconomic status (SES), 41.1% had poor WHR, and 39.7% had high-level inflammation. In total, 15,036 participants (39.9%) of the sample had a healthy diet, 7693 (20.4%) had HTN, 938 (2.5%) had DM, and 265 (0.7%) had stroke. Those with high-level inflammation were found to have higher likelihoods of being overweight (47.1% vs. 37.2%), having HTN (23.9% vs. 18.1%), DM (3.0% vs. 2.1%), and stroke (0.9% vs. 0.6%), as well as having low SES (52.7% vs. 48.1%) and an unhealthy diet (63.9% vs. 57.6%), compared with their counterparts.

**Figure 2:**
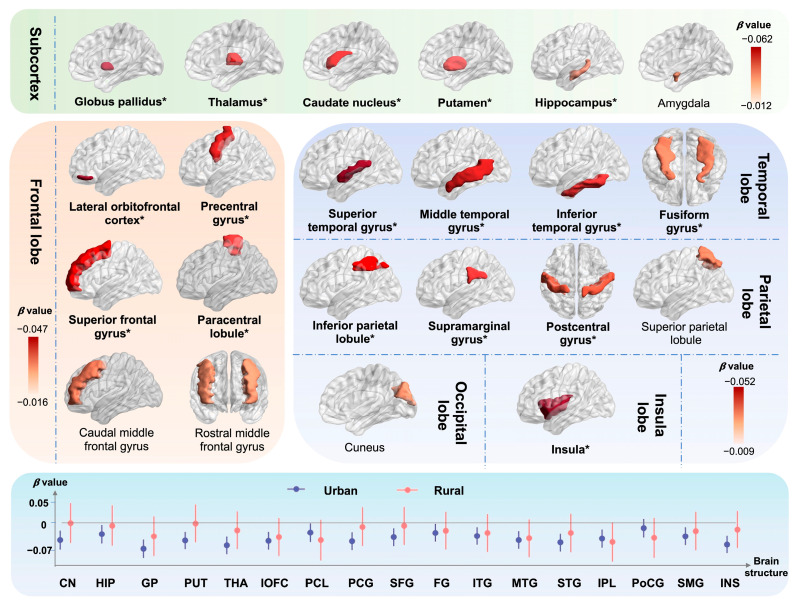
Associations between INFLA-score and brain imaging phenotypes stratified by urban/rural. The brain imaging phenotypes included the volumes of the cortex and subcortex. Among them, we showed specific cortical regions which involved frontal, temporal, and parietal lobes of the brain. The subcortical structures including volumes of amygdala, thalamus, caudate nucleus, hippocampus, putamen, and globus pallidus were presented. The associations between INFLA-score and brain structures were estimated among 37,699 participants with available neuroimaging data, utilizing multiple linear regression models. The result of urban-rural stratification analysis is shown at the bottom of the figure. Data were presented as the βs. Significance was determined through the FDR-corrected *p* value. Brain structure is in bold font and with an asterisk which indicates the negative association of statistical significance.

**Figure 3:**
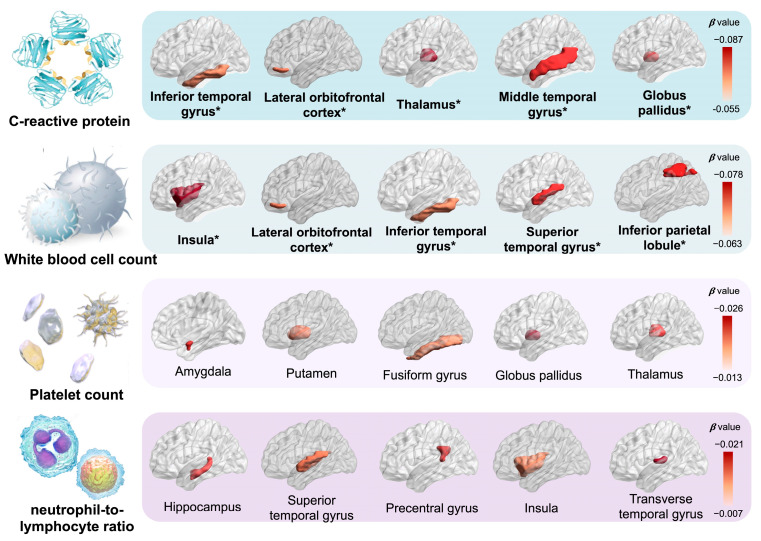
Associations between each component of INFLA-score and brain imaging phenotypes. The INFLA-score consisted of C-reactive protein, white blood cell, platelet counts, and neutrophil-to-lymphocyte ratio. The brain imaging phenotypes included the volumes of cortex and subcortex. Among them, we showed specific subcortical and cortical regions, which had large effect sizes. The associations between each component and brain structures were estimated utilizing multiple linear regression models. Data were presented as the βs. Significance was determined through the FDR-corrected *p* value. Brain structure is in bold font and with an asterisk which indicates the negative association of statistical significance.

The descriptive statistics for brain imaging phenotypes are demonstrated in Supplementary Table S4. The results of multiple linear regression analyses modelling the relation between LGI and brain structures by urban-rural are displayed in Figure 2 and Supplementary Tables S5 and S6. In general, with higher inflammation level, 6 out of 7 associations with volumes of subcortical structures and 20 out of 33 associations with volumes of cortical regions remained significant after FDR correction. Among all samples, we observed high inflammation as negatively related to the reduced volumes of the subcortex compared with the low level, including the globus pallidus (β [95% CI] = −0.062 [−0.083, −0.041]), thalamus (−0.053 [−0.073, −0.033]), caudate nucleus (−0.039 [−0.060, −0.017]), and putamen (−0.039 [−0.059, −0.019]), and others. As for cortical regions, high-level inflammation indicated a consistently hazardous effect on the atrophy of frontal, temporal, parietal, and insula lobes, especially the insula (−0.052 [−0.072, −0.032]), superior temporal gyrus (−0.049 [−0.069, −0.028]), lateral orbitofrontal cortex (−0.047 [−0.068, −0.027]), medial orbitofrontal cortex (−0.047 [−0.068, −0.027]), and others. Furthermore, we observed the most significant effects among urban residents, especially the globus pallidus (−0.069 [−0.092, −0.046]), thalamus (−0.060 [−0.082, −0.038]), medial orbitofrontal cortex (−0.049 [−0.071, −0.027]), and others. As shown in Figure 3 and Supplementary Tables S7 and S8, similar results were also observed for the associations of elevated CRP and white blood cell, the components of the INFLA-score, with brain imaging phenotypes, such as thalamus (CRP: -0.087 [−0.108, −0.065], white blood cell: −0.048 [−0.069, −0.027]), medial orbitofrontal cortex (CRP: −0.064 [−0.085, −0.043], white blood cell: −0.072 [−0.093, −0.051]), middle temporal gyrus (CRP: −0.060 [−0.081, −0.038], white blood cell: −0.055 [−0.076, −0.034]), precuneus (CRP: −0.039 [−0.061, −0.018], white blood cell: −0.054 [−0.075, −0.033]), insula (CRP: −0.055 [−0.076, −0.034], white blood cell: −0.078 [−0.098, −0.057]), and others.

**Figure 4:**
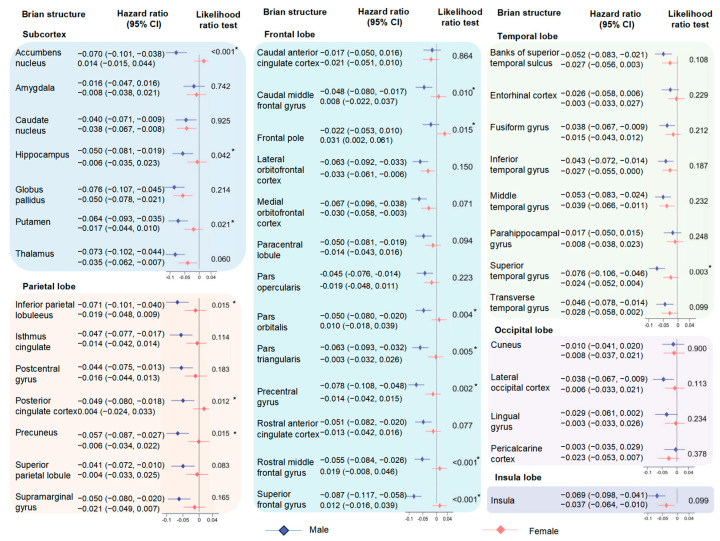
Associations between INFLA-score and brain imaging phenotypes by sex. The analyses stratified by sex (male/female) were performed utilizing multiple linear regression models. Dots with error bars colored in blue and red were HR (95% CI) for participants with males and female, respectively. The asterisk indicates the association of statistical significance.

**Figure 5:**
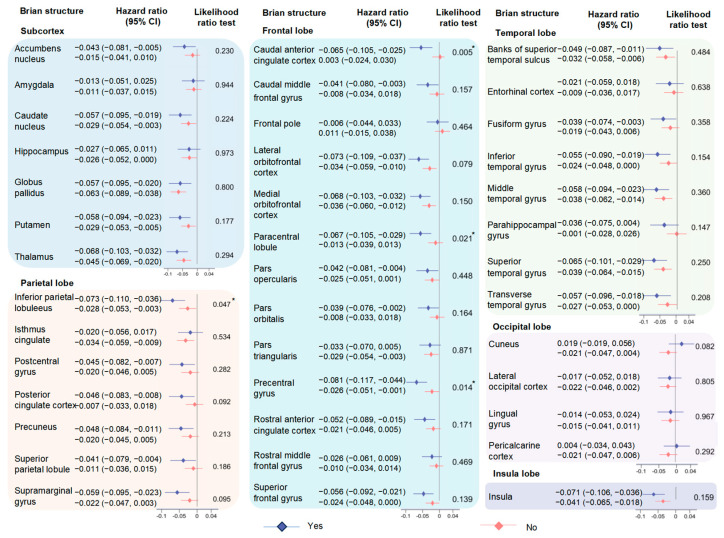
Associations between INFLA-score and brain imaging phenotypes by physical frailty. The analyses stratified by physical frailty (yes/no) were performed utilizing multiple linear regression models. Dots with error bars colored in blue and red were HR (95% CI) for participants with physical frailty and without physical frailty, respectively. The asterisk indicates the association of statistical significance.

When the subgroup analyses were stratified by gender, the associations between high inflammation and changes of brain structure in males were generally stronger than those in females (accumbens nucleus: −0.070 [−0.101, −0.038] for males, 0.014 [−0.015, 0.044] for females, *p* for interaction < 0.001; rostral middle frontal gyrus: −0.055 [−0.084, −0.026], 0.019 [−0.008, 0.046], *p* for interaction < 0.001; superior temporal gyrus: −0.076 [−0.106, −0.046], −0.024 [−0.052, 0.004], *p* for interaction = 0.013; inferior parietal lobule: −0.071 [−0.101, −0.040], −0.019 [−0.048, 0.009], *p* for interaction = 0.015; and others) (Figure 4). Moreover, the negative effect of high-level inflammation on the volumes of brain structures tended to be stronger in the participants with physical frailty (precentral gyrus: −0.081 [−0.117, −0.044] for yes, −0.026 [−0.051, −0.001] for no, *p* for interaction = 0.014); paracentral lobule: −0.067 [−0.105, −0.029], −0.013 [−0.039, 0.013], *p* for interaction = 0.021; and caudal anterior cingulate cortex: −0.065 [−0.105, −0.025], 0.003 [−0.024, 0.030], *p* for interaction = 0.005) (Figure 5). The results of age and sex stratifications between four components of the INFLA-score and brain structures are shown in Supplementary Tables S9–S12. However, few heterogeneities were observed among other subgroups, especially the WHR (*p* for interaction > 0.05) (Figures S2–S4). The results of the sensitivity analyses indicated that the main findings were robust (Supplementary Table S13).

## 4. Discussion

Our analysis was conducted to systematically explore the associations between LGI, considered as a chronic and subclinical inflammatory condition, and brain structural alteration. The results identified a significant inverse association between LGI and volumes of some brain gray matter phenotypes, especially globus pallidus, insula and lateral orbitofrontal cortex. These findings reinforce the idea that LGI may contribute to chronic diseases, such as neuropsychiatric diseases, among the middle and elderly population.

Epidemiological analyses reported that a high LGI level would increase risks of different chronic diseases and pathological conditions, such as adverse mental symptoms [9,20,28], neuropsychiatric diseases [21,29], cardiovascular diseases [30,31,32] and insulin resistance [33]. Despite the abundant literature data observed, the associations between LGI and some adverse health outcomes and subclinical states [9,20,21,22,28,30,31,32,33,34,35] with sample sizes ranging from approximately 200 to 10,000 individuals, our study extended the prior research by illustrating the association of LGI biomarkers with brain structural and organic alteration with a larger sample size. Our results revealed a link of LGI with atrophy of brain imaging phenotypes, in line with previous studies showing adverse associations between inflammatory biomarkers and volumes of hippocampal [36], left medial temporal lobe [37] or total brain [38,39]. Similarly, both cohort studies (based on multi-racial populations) and animal experiments (based on inducible mouse models of AD-like pathology) reported significant associations of inflammation with brain volume and cognitive decline [29,40]. Similar results were also observed in different study populations. Significantly negative associations between interleukin (IL)-6 cytokine levels and grey matter volumes were found in the Asian adult population, especially the anterior cingulate cortex [41]. Meanwhile, a study in the Czech Republic with severely obese individuals suggested the possibility of CRP to mediate the relationship between changes in visceral adiposity and the thickness of depression-related cortical areas [42]. Furthermore, a cross-sectional study, conducted in New York with middle-aged and older adults, showed that obesity-related inflammation reduces the integrity of the brain structure [43].

However, positive associations between inflammatory biomarkers, including CRP and WBC, and volumes of regional gray matter were observed in a cross-sectional study focused on children with overweight and obesity, mainly due to the fact that brain structure was particularly vulnerable to the effects of age [44]. A population-based retrospective cohort study with 308,352 participants illustrated that metabolic syndrome was significantly positive associated with anxiety risks by elevating the level of chronic inflammation [45]. However, in our subgroup analysis on metabolic syndrome, the synergetic effect of metabolic syndrome and LGI on brain morphological alteration is feeble, which may originate from rigorous study population exclusion criterion.

The key to linking inflammatory biomarkers to brain structural changes are the roles of distinct inflammatory markers in the neurodegenerative diseases process. Immunoreactivity of CRP in neurofibrillary tangles of Alzheimer’s disease evidenced that LGI was involved in neurodegenerative diseases progression [46]. Both increased and suppressed immune activity may increase psychiatric symptoms severity, as low levels of white blood cells indicate insufficient production and low immunity, and high levels of white blood cells may indicate an inflammatory state [47]. Detectable association of CRP smaller left medial temporal lobes as buttress verbal episodic memory consolidation hinted that LGI was related to cognitive decline [37]. In parallel, abnormal levels of CRP and white blood cell counts could cause an increase in Aβ accumulation and activated microglia (the brain’s major innate immune cells) [48], which may become a presumable mechanism for the effects inflammation on brain morphology. Nevertheless, it is still needed to illuminate the complicated mechanism that LGI used to lead to brain structural and functional alteration even in diseases.

**Figure 6:**
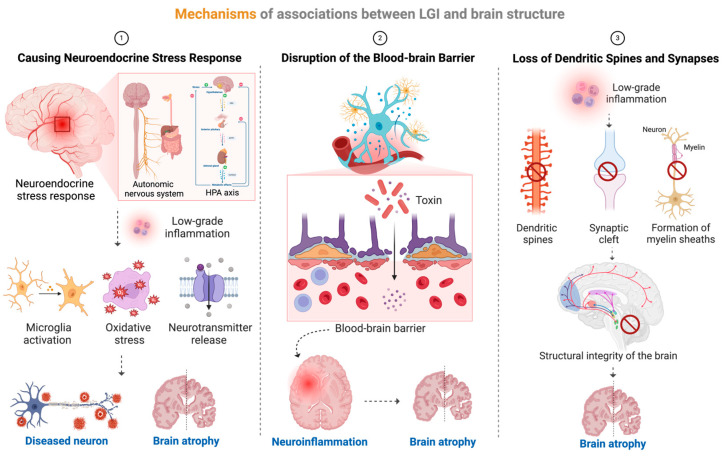
Mechanisms of associations between LGI and brain structure.

The associations between LGI and brain volume shrinkage involved complex biological processes, which might be interpreted in at least three different ways (Figure 6). First, neuroinflammation was chronically activated in the condition of LGI, which could damage neurons through microglia activation, oxidative stress, and excessive excitatory neurotransmitter (e.g., glutamate) release, leading to neuronal apoptosis and shrinkage of brain volume [49]. Second, inflammatory factors could increase the permeability of the blood–brain barrier, leading to the entry of toxins into the brain tissue, thus inducing neuroinflammation. Meanwhile, LGI impaired the function of cerebral blood vessels, reduced cerebral blood flow, affected the supply of oxygen and nutrients, and could cause damage to the endothelial cells of cerebral blood vessels and inflammation of blood vessel walls, all of which could lead to the death of brain cells and atrophy of the brain [40]. Finally, inflammatory factors inhibited the formation and remodeling of synapses between neurons, leading to the loss of dendritic spines and synapses, affecting the formation of myelin sheaths in the white matter of the brain, thus affecting the transmission of information and the structural integrity of the brain, and leading to shrinkage of the brain volume as well [50].

Chronic inflammation might begin in a slow, insidious, and even unnoticed manner, which was compatible with the temporal progression of numerous neuropsychiatric diseases. A longitudinal cohort analysis showing a reduction in the temporal, frontal, and parietal cortex in subjects with a clinically high risk for psychosis [51] was in keeping with decreased volumes in our results, thus indicating the role of inflammation on brain perception and mental activity. Evidence has also emerged for abnormalities in the frontal regions in patients with an autism spectrum disorder [52], which was compatible with our speculation that LGI affects cognitive function by altering brain frontal regions. In parallel, the thalamus and frontal lobe were strongly associated with cognitive–emotional processing, inhibiting fear responses [53] and atrophy of which in our study indicated inflammation affecting the brain cognitive and emotional function of individuals. As for mental health, inflammatory markers might contribute to the maintenance of fear- and anxiety-based symptoms by affecting the activity and connections of regions of the brain implicated in the etiology of some brain disorders, including the frontal and insular lobe [54]. Hence, associations between LGI and atrophy brain regions involving pathologic process or disease progression of various brain diseases, and damage in different regions may correspond to different symptoms of different diseases.

Moreover, our investigations take synergistic effects of some possible factors that are associations between INFLA-score and brain structural traits into consideration, and the differences were mainly observed among groups stratified by gender and physical frailty. In a large study of 765 patients with bipolar disorder aged 18–62 years from the United States, those with an abnormal white blood cell level showed more severe symptoms, especially among men [47], of which the gender difference may stem from the different effects of sex hormones. It was found that female sex hormones have a protective effect, while male sex hormones can suppress immune responses [55]. A prospective study with 314,998 participants showed that physical frailty was an independent risk factor of incident Parkinson diseases [56]. This is compatible with the dopamine imbalance hypothesis that physical frailty is related to dopamine imbalance, thus affecting brain structure and function [57]. Nevertheless, further studies are warranted to elucidate specific mechanisms of the observed between-group differences in associations of INFLA-score with brain morphological alteration.

Numerous literature data showed the detrimental or positive effect of special dietary consumption or medication use on LGI levels [19,58,59], which is evidence that LGI may serve as a crucial therapeutic target for treating those brain diseases in the future. As a mediator, LGI has been also shown to be involved in pathology of some chronic diseases led by various risk factors, such as body mass index [13], and inflammatory diet [22,28]. Differences in air quality [60], pro-inflammatory diet [61], and obesity factors [62] have been observed in rural and urban aeras. Specifically, urban residents experience poorer air quality than rural areas due to industry/combustion, the urban heat island effect, and road transport in urban areas [63]. Due to the urban lifestyle characterized by fast pace and increased shift work, dietary patterns of citizens have changed, encompassing irregular eating habits of fast food, breakfast omission and late-night snacking, resulting in increasing consumptions of the pro-inflammatory diet [62,64]. Obesity is listed as a vital factor contributing to the difference in rural and urban inflammation as a result of stimulation of cellular immune pathways in adipocytes [62]. Accordingly, it is necessary with urbanization to propose inflammatory intervention measures for urban populations to prevent chronic diseases, especially brain diseases.

However, there are some limitations to our analyses. First, the results cannot be generalized to the entire population as UK Biobank is not representative of the entire population and a healthy volunteer bias exists [65]. Second, the extensionality is an inevitable issue since the White European ancestry dominated the population in the UK Biobank (~97%). Our results about the associations between LGI and the shrinkage of multiple brain structural traits could be appropriate for the White European population so far, and studies in other populations, especially different racial types, are in high demand to validate the generalizability of results. Third, although INFLA-score is an essential indicator for integrating various inflammation biomarkers and evaluating the LGI, it ignored that those inflammation biomarkers were highly associated and influenced each other. The use of this metric may produce multi-collinearity and bias the final result. Fourth, we estimated the potential effects of individual LGI on brain structural traits with UK Biobank data. However, numerous disease onsets or progressions were more related to brain functional activities [51,54,66,67], which were not assessed in this study because of data inaccessibility. Further studies are in great demand to depict the whole picture of LGI on the human brain system by figuring out the effects of LGI on brain functional traits. Fifth, since the observational studies based on a single cohort could not infer causality and the required data were unavailable, multiple cohorts’ studies focused on different populations are needed to validate our results. Sixth, other inflammatory indicators that were not included in the INFLA-score, such as tumor necrosis factor-α, interleukin-1β or fibrinogen, were not taken account into evaluation of LGI due to the inaccessibility of those data in UK Biobank. Finally, variations beyond the normal ranges of each inflammation biomarker may lead to an overestimation of the corresponding risks, owing to some subjects under study with a clinical inflammatory sign or suffering from acute inflammation or certain hematological diseases. However, the bias was feeble when we excluded individuals with extreme levels of inflammation biomarkers.

## 5. Conclusions

To sum up, the conceptual and design framework of our investigation is to characterize the associations between LGI and brain imaging phenotypes, thus showing that LGI may lead to subclinical cognitive decline or neuropsychic diseases partly via structural neural pathways. Moreover, our analyses revealed that more significant associations of LGI with the atrophy of brain structure among male or individuals with physical frailty. These findings not only contribute to the evolvement of clinical diagnosis and therapy, but also provide a novel perspective for the development of new preventive strategies, namely, when brain lesions are subclinical and without any apparent clinical sign, inflammatory intervention, such as diet therapy, is an early preventive strategy. 

## Data Availability

All the data for this study will be made available upon reasonable request to the corresponding authors.

## Supplementary Materials

The following supporting information can be downloaded at: https://www.mdpi.com/article/10.3390/nu16142313/s1, eMethods [26,27,68,69,70]; Figure S1: Definition, exclusion and history of INFLA-score; Figure S2: Associations between INFLA-score and brain imaging phenotypes by age; Figure S3: Associations between INFLA-score and brain imaging phenotypes by Mets; Figure S4: Associations between INFLA-score and brain imaging phenotypes by WHR; Table S1: Calculation method of INFLA-score; Table S2: Definitions of brain phenotypes; Table S3: Baseline characteristics of participants grouped by INFLA-score; Table S4: Descriptive analysis of brain imaging phenotypes; Table S5: Main associations between INFLA-score and subcortex by residential region; Table S6: Main associations between INFLA-score and cortex by residential region; Table S7: Associations of CRP, WBC, PLT and NLR with subcortical regions; Table S8: Associations of CRP, WBC, PLT and NLR with cortical regions; Table S9: Associations of CRP and WBC with brain imaging phenotypes by age; Table S10: Associations of PLT and NLR with brain imaging phenotypes by age; Table S11: Associations of CRP and WBC with brain imaging phenotypes by sex; Table S12: Associations of PLT and NLR with brain imaging phenotypes by sex; Table S13: Sensitivity analysis of the main associations.
