# Supplementary material for: Chronic Low-Grade Inflammation and Brain Structure in the Middle-Aged and Elderly Adults
Source: Nutrients. 2024 Jul 18;16(14):2313. doi: 10.3390/nu16142313 (PMC11280392; doi:10.3390/nu16142313)
Supplement: Supplementary file 1 [file nutrients-16-02313-s001.zip › nutrients-3092123-supplementary.pdf]

Supplementary Information

Chronic Low-Grade Inflammation and Brain Structure in the Middle-Aged and Elderly Adults

eMethods 2

Figure S1. Definition, exclusion and history of INFLA-score 4

Figure S2. Associations between INFLA-score and brain imaging phenotypes by age 5

Figure S3. Associations between INFLA-score and brain imaging phenotypes by WHR 6

Figure S4. Associations between INFLA-score and brain imaging phenotypes by MetS 7

Table S1. Calculation method of INFLA-score 8

Table S2. Definitions of brain phenotypes 9

Table S3. Baseline characteristics of participants grouped by INFLA-score 10

Table S4. Descriptive analysis of brain imaging phenotypes 11

Table S5. Main associations between INFLA-score and subcortex by residential region 12

Table S6. Main associations between INFLA-score and cortex by residential region 13

Table S7. Associations of CRP, WBC, PLT and NLR with subcortical regions 14

Table S8. Associations of CRP, WBC, PLT and NLR with cortical regions 15

Table S9. Associations of CRP and WBC with brain imaging phenotypes by age 17

Table S10. Associations of PLT and NLR with brain imaging phenotypes by age 19

Table S11. Associations of CRP and WBC with brain imaging phenotypes by sex 21

Table S12. Associations of PLT and NLR with brain imaging phenotypes by sex 23

Table S13. Sensitivity analysis of the main associations 25

eMethods

Healthy lifestyles

Four recognized healthy lifestyle factors, including smoking status, regular physical activity, healthy sleep pattern and healthy diet, were selected. Baseline data was assessed using an in-person touchscreen questionnaire at the UK Biobank centres. Among them, smoking status of participants was categorized as never smoking or current smoking (Data field: 20116). Regular physical activity was defined as the following criteria: at least 150 minutes of moderate activity per week, 75 minutes of vigorous activity or equivalent combination [68] (Data field: 22035). Healthy sleep score was generated based on five sleep behaviors (chronotype, duration, insomnia, snoring, and excessive daytime sleepiness) [69, 70]. Five aspects of healthy sleep were defined as follows: early chronotype ("morning" or "morning than evening") (Data field: 1180); sleep 7-8 h per day (Data field: 1160); reported never or rarely insomnia symptoms (Data field: 1200); no self-reported snoring (Data field: 1210); and no frequent daytime sleepiness ("never/rarely" or "sometimes") (Data field: 1220). A healthy sleep pattern can be defined as fulfilling the four or more aspects above. For diet, a healthy diet was defined based on intake of at least 4 of 7 commonly food groups recommended as dietary priorities for cardiometabolic health. The frequencies of each healthy diet component are as follows: ≥3 servings/day for fruits; ≥3 servings/day for vegetables; ≥2 servings/week for fish; ≤1 serving/week processed meats; ≤1.5 servings/week for unprocessed red meats; ≥ 3 servings/day for whole grains; ≤1.5 servings/day for refined grains. (Data field: 1309, 1319, 1289, 1299, 1329, 1339, 1349, 1369, 1379, 1389, 1438, 1448, 1458, 1468)

Physical frailty

The original definition of the physical frailty phenotypes were described and applied in the Cardiovascular Health Study [71], and the items have been adapted in the UK Biobank. Weight loss (Data field: 2306) was self-reported as the question "Compared with one year ago, has your weight changed?" (response: yes, lost weight =1; others =0). Exhaustion (Data field: 2080) was self-reported according to the question "Over the past 2 weeks, how often have you felt tired or had little energy?" (response: more than half the days or nearly every day =1; others =0). Physical activity (Data field: 31, 21022, 22037, 22038, 22039) was assessed using the International Physical Activity Questionnaire short form, which computed the sum of walking, moderate activity, and vigorous activity to estimate the total metabolic equivalents (MET) minutes per week. Physical activity was categorized into

quintiles of sex- and age-specific levels of total MET minutes per week, in which the lowest quintile was identified as “low physical activity.” Slow gait speed (Data field: 924) was self-reported with the question “How would you describe your usual walking pace?” (response: slow =1; others =0). Hand grip strength (Data field: 31, 21001, 46, 47) was measured by using a Jamar J00105 hydraulic hand dynamometer. The measured grip strength was expressed in kilograms by sex- and BMI-adjusted, and the cutoff points have referenced the points by Fried and colleagues [71]. Participants were classified as frail (fulfilled ≥3 criteria), pre-frail (fulfilled 1–2 criteria), or non-frail (fulfilled 0 criteria).

Metabolic syndrome

Metabolic syndrome (MetS) was defined based on the harmonized criteria that meeting the three or more of the following: elevated waist circumference, triglycerides, blood pressure, blood glucose and CRP, as well as reduced high density lipoprotein cholesterol [72]. In detail, the established reference values for women (≥88 cm) and men (≥102 cm) were used as cutoff points for defining unhealthy waist circumference (Data field: 8). The elevated triglyceride was defined as triglyceride levels of 150 mg/dL (Data field: 30870). The elevated blood pressure was defined as a systolic blood pressure ≥130 mmHg, a diastolic blood pressure ≥85 mmHg or antihypertensive medication use (Data field: 4080, 4079, 93, 94, 6177). And the elevated CRP was defined as CRP level ≥3.0 mg/L (Data field: 30710).

Reference:

68. Lloyd-Jones, D.M.; Hong, Y.; Labarthe, D.; Mozaffarian, D.; Appel, L.J.; Van Horn, L.; Greenlund, K.; Daniels, S.; Nichol, G.; Tomaselli, G.F., et al. *Defining and setting national goals for cardiovascular health promotion and disease reduction: the American Heart Association's strategic Impact Goal through 2020 and beyond.* Circulation, 2010. **121**(4): p. 586-613.
69. Fan, M.; Sun, D.; Zhou, T.; Heianza, Y.; Lv, J.; Li, L. and Qi, L. *Sleep patterns, genetic susceptibility, and incident cardiovascular disease: a prospective study of 385 292 UK biobank participants.* Eur Heart J, 2020. **41**(11): p. 1182-1189.
70. Li, X.; Xue, Q.; Wang, M.; Zhou, T.; Ma, H.; Heianza, Y. and Qi, L. *Adherence to a Healthy Sleep Pattern and Incident Heart Failure: A Prospective Study of 408 802 UK Biobank Participants.* Circulation, 2021. **143**(1): p. 97-99.
71. LP., F.; CM., T.; J., W.; C., N.A.H.; J., G.; T., S.; R., T.; WJ., K.; G., B. and MA., M. *Frailty in older adults: evidence for a phenotype.* J Gerontol A Biol Sci Med Sci, 2001. **56**(3): p. M146-56.
72. Dregan, A.; Rayner, L.; Davis, K.A.S.; Bakolis, I.; Arias de la Torre, J.; Das-Munshi, J.; Hatch, S.L.; Stewart, R. and Hotopf, M. *Associations Between Depression, Arterial Stiffness, and Metabolic Syndrome Among Adults in the UK Biobank Population Study: A Mediation Analysis.* JAMA Psychiatry, 2020. **77**(6): p. 598-606.

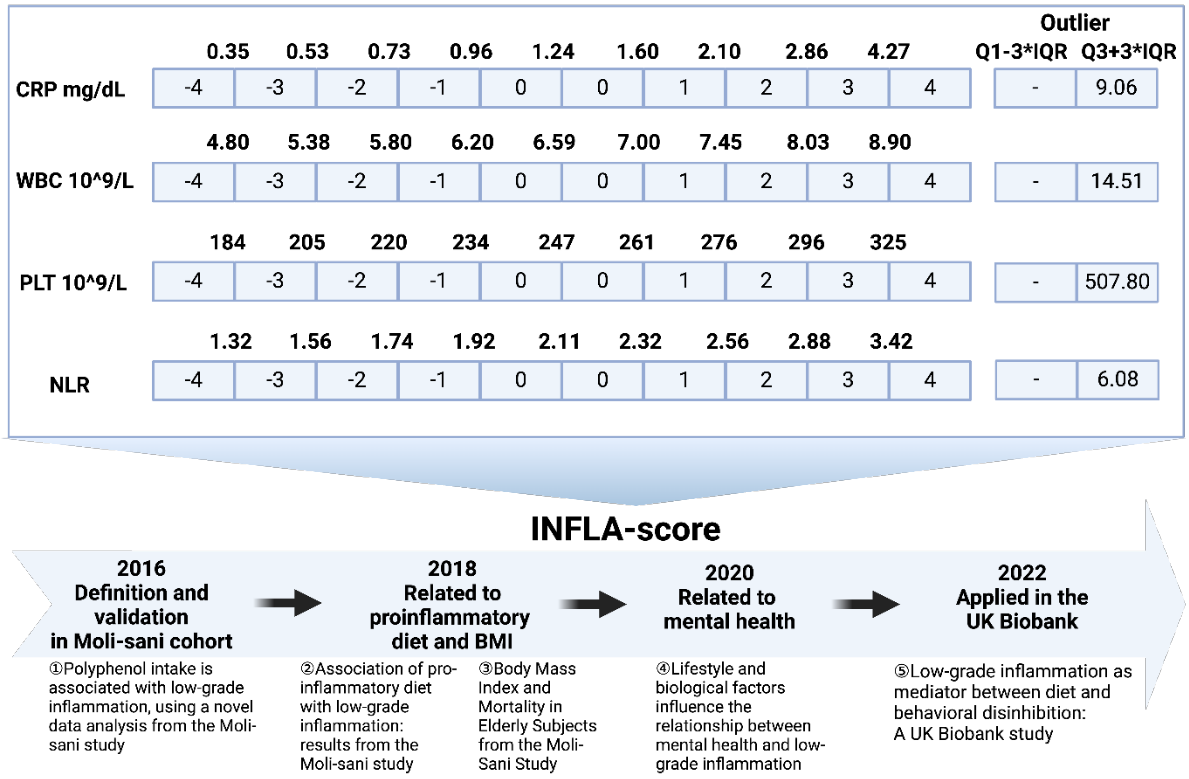

Figure S1. Definition, exclusion and history of INFLA-score

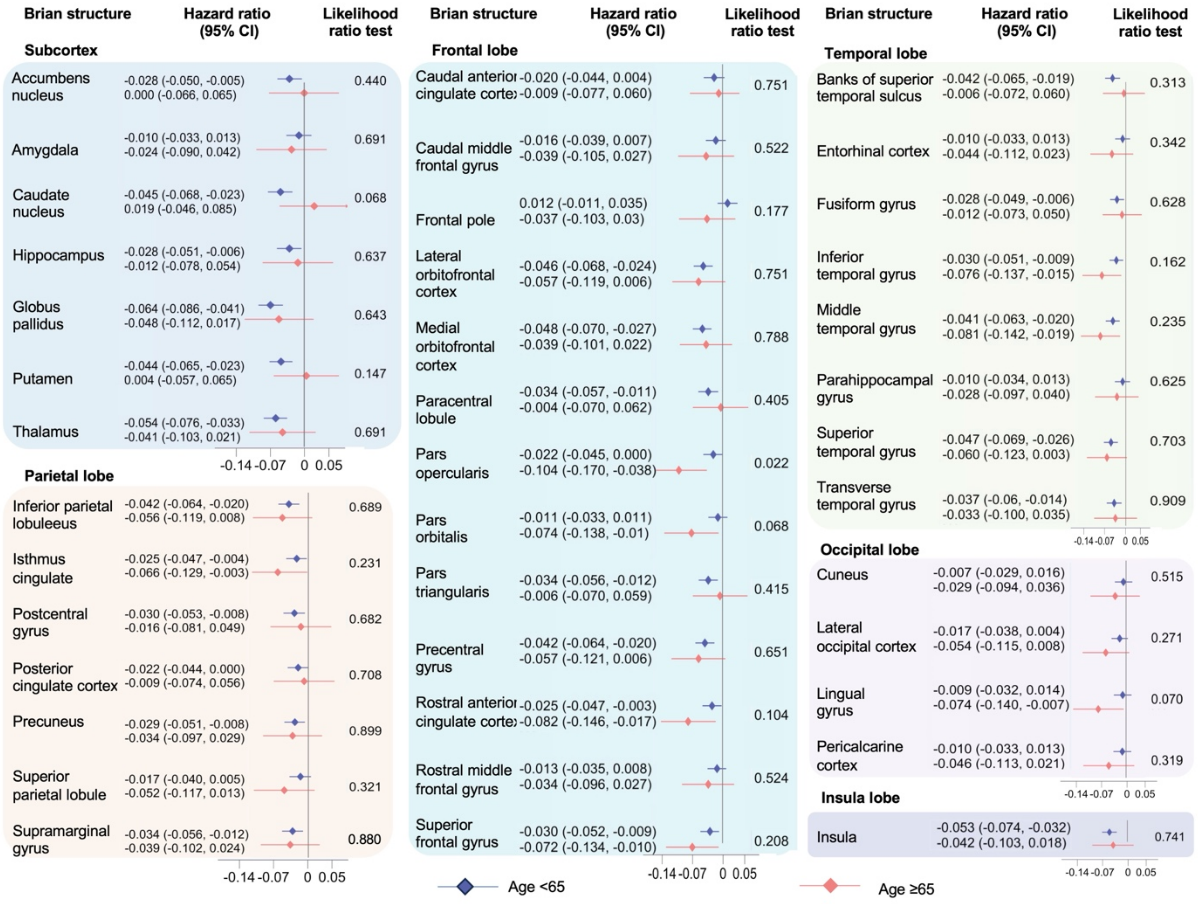

Figure S2. Associations between INFLA-score and brain imaging phenotypes by age

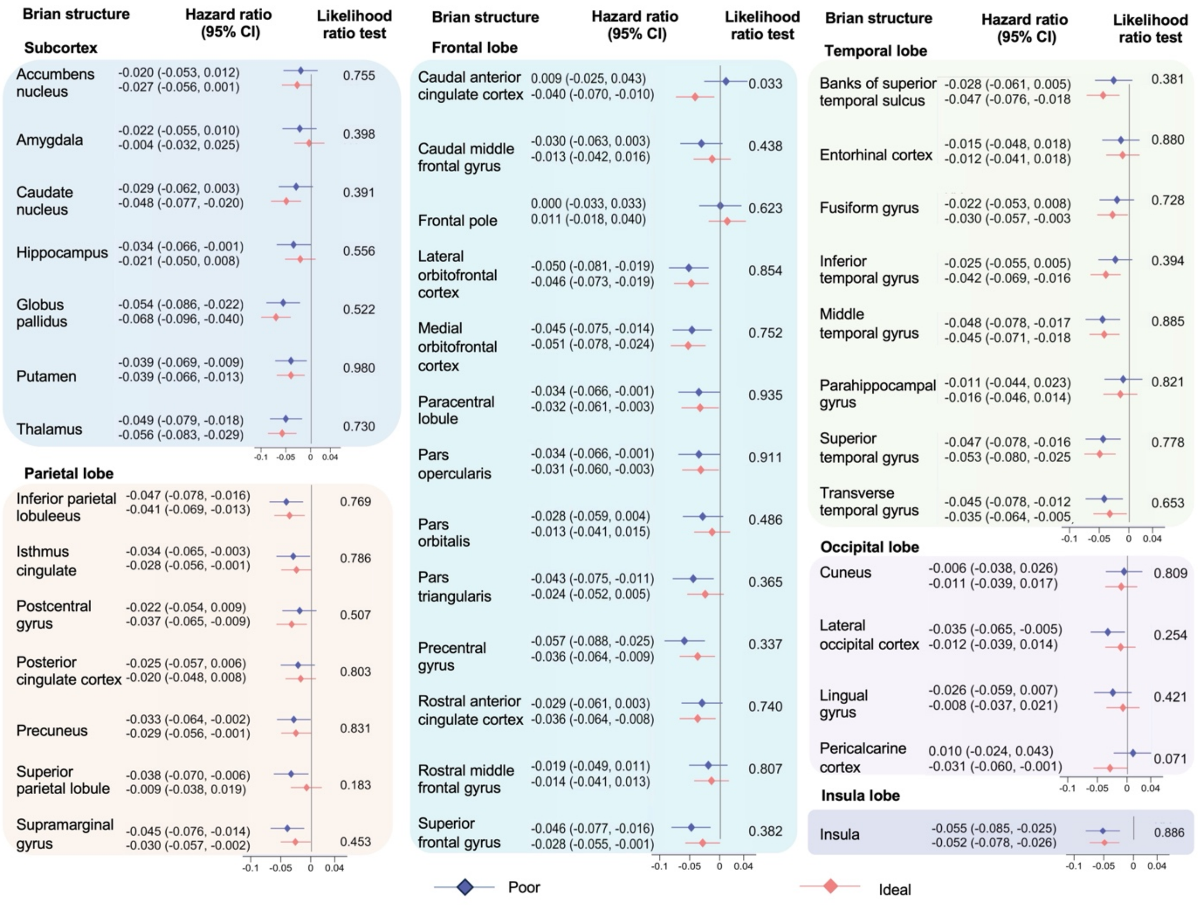

Figure S3. Associations between INFLA-score and brain imaging phenotypes by WHR

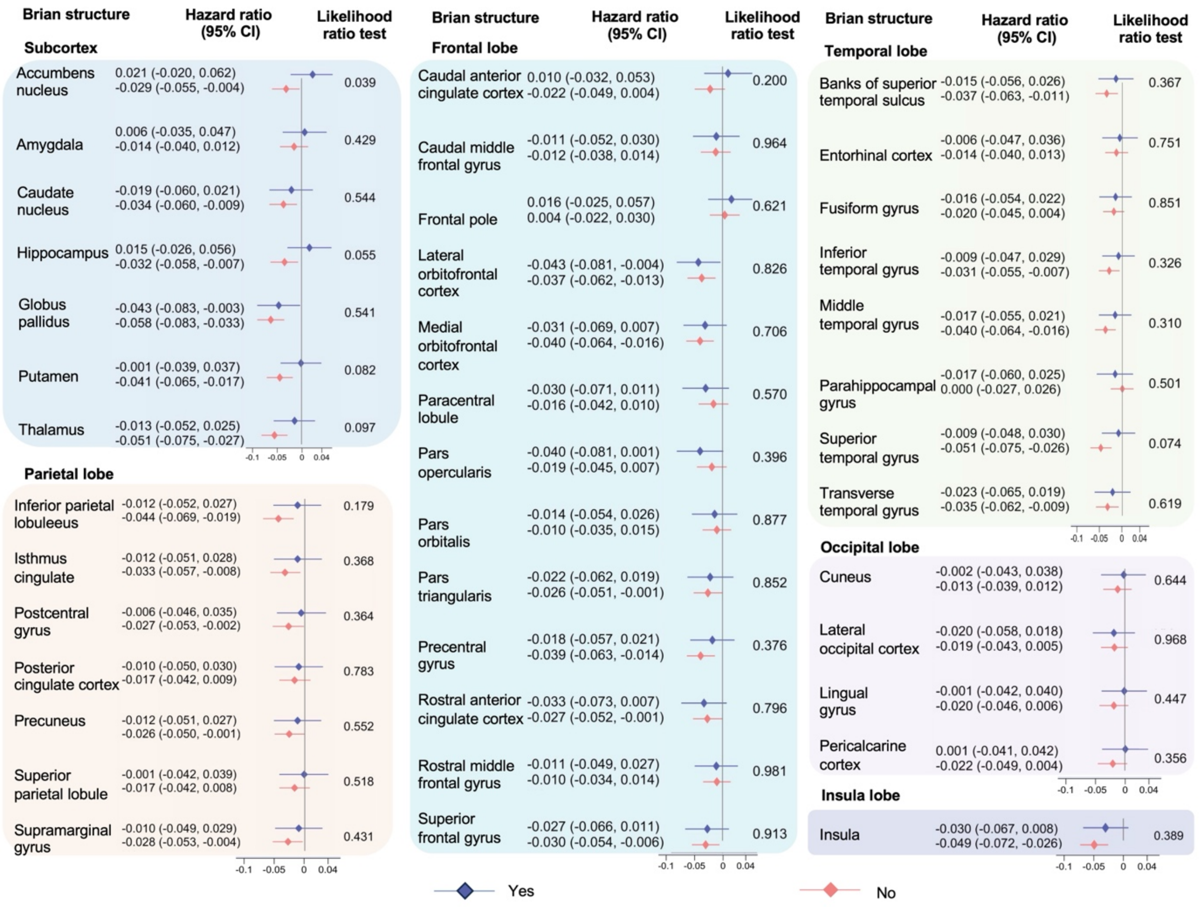

Figure S4. Associations between INFLA-score and brain imaging phenotypes by MetS

Table S1. Calculation method of INFLA-score

Components of INFLA-score[a]	UKB data field	Field code	Score of each biomarker level									
			Q1	Q2	Q3	Q4	Q5	Q6	Q7	Q8	Q9	Q10
C-reactive protein	/	30710	-4	-3	-2	-1	0	1	2	3	4	5
White blood cell (leukocyte) count	/	30000	-4	-3	-2	-1	0	1	2	3	4	5
Platelet count	/	30080	-4	-3	-2	-1	0	1	2	3	4	5
neutrophil-to-lymphocyte ratio (NLR)	neutrophil count	30140	-4	-3	-2	-1	0	1	2	3	4	5
	lymphocyte count	30120										

a The INFLA-score is measured in four dimensions (C-reactive protein, white blood cell, platelet counts, and neutrophil-to-lymphocyte ratio), ranging from -16 to 16. Q1-Q10 are the deciles of biomarker levels such as CRP, WBC, PLT, NLR. Each biomarker level is scored using this scoring system, and the scores are summarized to calculate the INFLA score.

Table S2. Definitions of brain phenotypes

**Brain phenotypes**	**Entity (Abbr)**
Subcortical structure	accumbens nucleus (NAc)
	amygdala (AMYG)
	caudate nucleus (CN)
	hippocampus (HIP)
	globus pallidus (GP)
	putamen (PUT)
	thalamus (THA)
Cortical structure	
Frontal lobe	caudal anterior cingulate cortex (cACC)
	caudal middle frontal gyrus (cMFG)
	frontal pole (FP)
	lateral orbitofrontal cortex (lOFC)
	medial orbitofrontal cortex (mOFC)
	paracentral lobule (PCL)
	pars opercularis (OP)
	pars orbitalis (ORB)
	pars triangularis (TRI)
	precentral gyrus (PCG)
	rostral anterior cingulate cortex (rACC)
	rostral middle frontal gyrus (RMFG)
	superior frontal gyrus (SFG)
Temporal lobe	banks of superior temporal sulcus (Bankssts)
	entorhinal cortex (EC)
	fusiform gyrus (FG)
	inferior temporal gyrus (ITG)
	middle temporal gyrus (MTG)
	parahippocampal gyrus (PHG)
	superior temporal gyrus (STG)
	transverse temporal gyrus (TTG)
Parietal lobe	inferior parietal lobule (IPL)
	isthmus cingulate (Isthmus)
	postcentral gyrus (PoCG)
	posterior cingulate cortex (PCC)
	precuneus (PCUN)
	superior parietal lobule (SPL)
	supramarginal gyrus (SMG)
Occipital lobe	cuneus (CUN)
	lateral occipital cortex (LOC)
	lingual gyrus (LG)
	pericalcarine cortex (PCAL)
Insula lobe	insula (INS)

Table S3. Baseline characteristics of participants grouped by INFLA-score

Characteristic	All (N=37699)	Low[a] (N=22721)	High (N=14978)	*P*.overall
**Age, year**				0.092
<65	33781 (89.6%)	20409 (89.8%)	13372 (89.3%)	
≥65	3918 (10.4%)	2312 (10.2%)	1606 (10.7%)	
**Sex**				<0.001
Female	20015 (53.1%)	11428 (50.3%)	8587 (57.3%)	
Male	17684 (46.9%)	11293 (49.7%)	6391 (42.7%)	
**IMD**				<0.001
Low	18870 (50.1%)	11783 (51.9%)	7087 (47.3%)	
High	18829 (49.9%)	10938 (48.1%)	7891 (52.7%)	
**WHR**[b]				<0.001
Ideal	22195 (58.9%)	14272 (62.8%)	7923 (52.9%)	
Poor	15504 (41.1%)	8449 (37.2%)	7055 (47.1%)	
**Lifestyle**				
Never smoking[c]	35366 (94.0%)	21591 (95.2%)	13775 (92.1%)	<0.001
Never drinking[d]	1647 (4.4%)	950 (4.2%)	697 (4.7%)	0.030
Regular physical activity[e]	17679 (54.7%)	11233 (57.4%)	6446 (50.7%)	<0.001
Healthy sleep pattern[f]	21690 (57.5%)	13378 (58.9%)	8312 (55.5%)	<0.001
Healthy diet[g]	15036 (39.9%)	9623 (42.4%)	5413 (36.1%)	<0.001
**Prevalence**				
Hypertension	7693 (20.4%)	4114 (18.1%)	3579 (23.9%)	<0.001
Diabetes	938 (2.5%)	486 (2.1%)	452 (3.0%)	<0.001
Stroke	265 (0.7%)	130 (0.6%)	135 (0.9%)	<0.001

Abbreviations: IMD, indices of multiple deprivation; WHR, waist to hip ratio.

a INFLA-score was categorized into levels of low (≤0) and high (>0).

b WHR was calculated as waist circumference (centimeter) divided by hip circumference (centimeter) and categorized into ideal (<0.9 for men and <0.85 for women) and poor (≥0.9 for men and ≥0.85 for women).

c Never smoking was required by questionnaire and categorized into yes (never smokers) and no (current or previous smokers).

d Never drinking was required by questionnaire and categorized into yes (never drinkers) and no (current or previous drinkers).

e Regular physical activity: ≥ 150 minutes of moderate-intensity per week or ≥ 75 minutes of vigorous-intensity per week, or a combination of both.

f Healthy sleep pattern was measured by 5 dimensions of sleep behaviors: early chronotype, sleep 7-8 hours/day, never/rarely or sometimes insomnia, no self-reported snoring, and never/rarely or sometimes excessive daytime sleepiness, and categorized into yes (≥4 healthy components) and no.

g Healthy diet was measured by seven healthy diet components: Fruits: ≥ 3 servings/day; Vegetables: ≥ 3 servings/day; Fish: ≥2 servings/week; Processed meats: ≤ 1 serving/week; Unprocessed red meats: ≤ 1.5 servings/week; Whole grains: ≥ 3 servings/day; Refined grains: ≤1.5servings/day, and categorized into yes (participants had ≥4 healthy diet components) and no.

Table S4. Descriptive analysis of brain imaging phenotypes

Brain imaging phenotypes[a]	P25	P50	P75	Mean	SD
**Subcortical phenotypes**					
NAc	367.5	437.5	507.5	438.1	104.4
AMYG	1096.5	1239.0	1385.0	1243.7	215.3
CN	3176.0	3444.0	3730.0	3464.4	415.4
HIP	3546.5	3829.0	4111.5	3827.5	435.6
GP	1622.0	1757.0	1906.0	1772.2	220.8
PUT	4389.0	4756.5	5151.0	4781.0	568.5
THA	7136.5	7611.0	8109.5	7639.5	724.8
**Cortical phenotypes**					
**Frontal lobe**					
cACC	1605.5	1855.0	2118.0	1871.9	397.4
cMFG	5840.0	6470.0	7138.5	6522.4	976.5
FP	984.0	1067.5	1157.5	1074.9	131.6
lOFC	7466.0	8019.5	8605.5	8055.3	839.8
mOFC	5570.5	5979.0	6430.5	6016.4	635.1
PCL	3675.3	3987.0	4327.0	4016.2	492.0
OP	4077.5	4453.5	4877.5	4503.3	609.1
ORB	2451.0	2661.5	2888.5	2678.9	327.9
TRI	3697.0	4057.0	4467.5	4102.4	570.0
PCG	13011.0	14029.5	15125.3	14093.8	1580.2
rACC	2234.0	2505.5	2807.0	2532.5	430.3
RMFG	14684.5	16033.0	17560.5	16194.5	2141.9
SFG	21261.0	23014.0	24924.3	23194.5	2716.0
**Parietal lobe**					
Bankssts	2184.5	2399.0	2639.5	2423.5	345.4
EC	1670.5	1873.5	2101.0	1898.9	329.1
FG	9404.5	10195.0	11045.5	10255.4	1225.4
ITG	10600.5	11578.5	12643.5	11667.3	1511.4
MTG	10807.5	11784.0	12845.0	11867.5	1500.0
PHG	1809.0	1970.5	2141.5	1981.1	253.3
STG	11522.5	12470.0	13474.5	12542.4	1447.7
TTG	998.0	1110.0	1238.0	1125.5	179.5
**Parietal lobe**					
IPL	12525.5	13693.0	14989.0	13805.7	1828.8
isthmus cingulate	2405.5	2646.0	2920.0	2677.9	386.2
PoCG	9219.5	10009.5	10875.5	10079.4	1237.6
PCC	3088.5	3376.5	3695.0	3398.2	459.0
PCUN	9640.0	10468.0	11360.0	10539.4	1273.6
SPL	12823.8	13916.5	15102.3	13996.2	1680.8
SMG	10356.3	11352.5	12457.5	11453.4	1561.8
**Occipital lobe**					
CUN	2814.5	3137.0	3508.5	3186.9	524.2
LOC	11495.5	12580.5	13756.5	12676.8	1669.6
LG	5898.0	6586.5	7328.0	6634.3	1053.8
PCAL	1952.0	2215.5	2527.5	2260.3	433.2
**Insula lobe**					
INS	6796.5	7308.5	7868.8	7359.5	790.7

Abbreviations: SD, standard deviation.

a The abbreviations of the phenotypes were shown in Table S2.

Table S5. Main associations between INFLA-score and subcortex by residential region

Brain structure[a]	Main association		Subgroup analysis	
			Urban population	Rural population
	β per IQR[b]	*P* value FDR[b]	β per IQR[b]	β per IQR[b]
NAc	-0.025 (-0.046, -0.003)	0.036	-0.026 (-0.050, -0.003)	-0.028 (-0.081, 0.024)
AMYG	-0.012 (-0.033, 0.010)	0.305	-0.019 (-0.042, 0.005)	0.031 (-0.022, 0.083)
CN	-0.039 (-0.060, -0.017)	0.002	-0.046 (-0.070, -0.022)	-0.001 (-0.053, 0.051)
HIP	-0.027 (-0.048, -0.005)	0.025	-0.030 (-0.054, -0.007)	-0.008 (-0.060, 0.045)
GP	-0.062 (-0.083, -0.041)	<0.001	-0.069 (-0.092, -0.046)	-0.036 (-0.087, 0.016)
PUT	-0.039 (-0.059, -0.019)	0.001	-0.047 (-0.069, -0.025)	-0.002 (-0.051, 0.047)
THA	-0.053 (-0.073, -0.033)	<0.001	-0.060 (-0.082, -0.038)	-0.020 (-0.069, 0.029)

Abbreviations: IQR, interquartile range; FDR, false discovery rate.

a The abbreviations of the phenotypes were shown in Table S2.

b The '*P* value FDR' is a measure of the error detection rate obtained by correcting for the difference significance *P* value. A low '*P* value FDR value' (typically less than 0.05) suggests statistical significance.

Table S6. Main associations between INFLA-score and cortex by residential region

Brain structure[a]	Main association		Subgroup analysis	
			Urban population	Rural population
	β per IQR[b]	*P* value FDR[b]	*β* per IQR[b]	*β* per IQR[b]
**Frontal lobe**				
cACC	-0.019 (-0.042, 0.003)	0.122	-0.020 (-0.045, 0.005)	-0.010 (-0.065, 0.044)
cMFG	-0.019 (-0.040, 0.003)	0.122	-0.028 (-0.052, -0.004)	0.030 (-0.023, 0.083)
FP	0.007 (-0.015, 0.028)	0.550	0.012 (-0.012, 0.036)	-0.015 (-0.068, 0.038)
lOFC	-0.047 (-0.068, -0.027)	<0.001	-0.048 (-0.070, -0.025)	-0.038 (-0.087, 0.012)
mOFC	-0.047 (-0.068, -0.027)	<0.001	-0.049 (-0.071, -0.027)	-0.036 (-0.086, 0.013)
PCL	-0.031 (-0.052, -0.009)	0.010	-0.026 (-0.050, -0.003)	-0.046 (-0.099, 0.007)
OP	-0.031 (-0.053, -0.009)	0.010	-0.033 (-0.056, -0.009)	-0.022 (-0.075, 0.030)
ORB	-0.018 (-0.039, 0.003)	0.122	-0.018 (-0.041, 0.005)	-0.016 (-0.068, 0.035)
TRI	-0.031 (-0.052, -0.010)	0.009	-0.035 (-0.059, -0.012)	-0.007 (-0.059, 0.044)
PCG	-0.044 (-0.064, -0.023)	<0.001	-0.049 (-0.071, -0.026)	-0.011 (-0.061, 0.040)
rACC	-0.031 (-0.052, -0.010)	0.009	-0.029 (-0.052, -0.005)	-0.033 (-0.085, 0.019)
RMFG	-0.016 (-0.036, 0.004)	0.153	-0.017 (-0.039, 0.004)	-0.004 (-0.053, 0.044)
SFG	-0.035 (-0.055, -0.014)	0.002	-0.038 (-0.061, -0.016)	-0.008 (-0.058, 0.041)
**Temporal lobe**				
Bankssts	-0.039 (-0.060, -0.017)	0.002	-0.041 (-0.065, -0.018)	-0.019 (-0.072, 0.033)
EC	-0.014 (-0.036, 0.008)	0.251	-0.020 (-0.044, 0.004)	0.015 (-0.039, 0.068)
FG	-0.026 (-0.046, -0.006)	0.020	-0.027 (-0.049, -0.004)	-0.021 (-0.070, 0.028)
ITG	-0.035 (-0.055, -0.014)	0.002	-0.035 (-0.057, -0.013)	-0.027 (-0.076, 0.022)
MTG	-0.046 (-0.066, -0.025)	<0.001	-0.046 (-0.068, -0.023)	-0.041 (-0.090, 0.008)
PHG	-0.012 (-0.035, 0.010)	0.305	-0.015 (-0.040, 0.010)	0.012 (-0.042, 0.067)
STG	-0.049 (-0.069, -0.028)	<0.001	-0.052 (-0.075, -0.030)	-0.027 (-0.077, 0.023)
TTG	-0.036 (-0.058, -0.014)	0.003	-0.037 (-0.061, -0.013)	-0.029 (-0.083, 0.024)
**Parietal lobe**				
IPL	-0.043 (-0.064, -0.023)	<0.001	-0.042 (-0.065, -0.019)	-0.051 (-0.102, 0.000)
isthmus cingulate	-0.030 (-0.050, -0.009)	0.010	-0.030 (-0.053, -0.007)	-0.018 (-0.068, 0.033)
PoCG	-0.029 (-0.050, -0.008)	0.013	-0.029 (-0.052, -0.006)	-0.027 (-0.079, 0.024)
PCC	-0.021 (-0.042, 0.001)	0.078	-0.014 (-0.038, 0.009)	-0.040 (-0.092, 0.012)
PCUN	-0.030 (-0.051, -0.009)	0.010	-0.033 (-0.056, -0.010)	-0.006 (-0.056, 0.044)
SPL	-0.021 (-0.042, 0.000)	0.077	-0.025 (-0.049, -0.002)	0.002 (-0.050, 0.054)
SMG	-0.035 (-0.055, -0.014)	0.003	-0.036 (-0.058, -0.013)	-0.022 (-0.072, 0.028)
**Occipital lobe**				
CUN	-0.009 (-0.030, 0.012)	0.419	-0.013 (-0.036, 0.011)	0.008 (-0.044, 0.060)
LOC	-0.021 (-0.041, -0.001)	0.057	-0.026 (-0.048, -0.004)	0.003 (-0.046, 0.052)
LG	-0.016 (-0.037, 0.006)	0.192	-0.019 (-0.043, 0.004)	0.013 (-0.040, 0.066)
PCAL	-0.014 (-0.036, 0.008)	0.251	-0.013 (-0.038, 0.011)	-0.006 (-0.060, 0.047)
**Insula lobe**				
INS	-0.052 (-0.072, -0.032)	<0.001	-0.058 (-0.079, -0.036)	-0.018 (-0.066, 0.030)

Abbreviations: IQR, interquartile range; FDR, false discovery rate.

a The abbreviations of the phenotypes were shown in Table S2.

b The '*P* value FDR' is a measure of the error detection rate obtained by correcting for the difference significance *P* value. A low '*P* value FDR value' (typically less than 0.05) suggests statistical significance.

Table S7. Associations of CRP, WBC, PLT and NLR with subcortical regions

Brain structure[a]	CRP		WBC		PLT		NLR	
	***β* per IQR[b]**	***P* value FDR[b]**	***β* per IQR[b]**	***P* value FDR[b]**	***β* per IQR[b]**	***P* value FDR[b]**	***β* per IQR[b]**	***P* value FDR[b]**
NAc	-0.054 (-0.077, -0.032)	<0.001	-0.027 (-0.049, -0.005)	0.018	-0.006 (-0.028, 0.015)	0.804	0.004 (-0.018, 0.025)	0.988
AMYG	-0.003 (-0.026, 0.020)	0.821	-0.012 (-0.034, 0.011)	0.324	-0.026 (-0.048, -0.004)	0.405	0.002 (-0.020, 0.023)	0.988
CN	-0.051 (-0.073, -0.028)	<0.001	-0.059 (-0.081, -0.037)	<0.001	-0.001 (-0.023, 0.021)	0.964	0.012 (-0.009, 0.034)	0.988
HIP	-0.045 (-0.067, -0.022)	<0.001	-0.016 (-0.038, 0.006)	0.172	-0.012 (-0.034, 0.009)	0.793	-0.014 (-0.035, 0.007)	0.988
GP	-0.086 (-0.108, -0.063)	<0.001	-0.051 (-0.073, -0.030)	<0.001	-0.025 (-0.047, -0.004)	0.405	0.002 (-0.019, 0.023)	0.988
PUT	-0.051 (-0.073, -0.030)	<0.001	-0.056 (-0.077, -0.035)	<0.001	-0.014 (-0.035, 0.006)	0.793	0.017 (-0.003, 0.036)	0.832
THA	-0.087 (-0.108, -0.065)	<0.001	-0.048 (-0.069, -0.027)	<0.001	-0.018 (-0.039, 0.002)	0.628	0.003 (-0.017, 0.023)	0.988

Abbreviations: IQR, interquartile range; FDR, false discovery rate; CRP, C-reactive protein; WBC, white blood cell; PLT, platelet counts; NLR, neutrophil-to-lymphocyte ratio.

a The abbreviations of the phenotypes were shown in Table S2.

b The '*P* value FDR' is a measure of the error detection rate obtained by correcting for the difference significance *P* value. A low '*P* value FDR value' (typically less than 0.05) suggests statistical significance.

Table S8. Associations of CRP, WBC, PLT and NLR with cortical regions

Brain structure[a]	CRP		WBC		PLT		NLR	
	β per IQR[b]	*P* value FDR[b]	β per IQR[b]	*P* value FDR[b]	β per IQR[b]	*P* value FDR[b]	β per IQR[b]	*P* value FDR[b]
**Frontal lobe**								
cACC	-0.025 (-0.049, -0.001)	0.050	-0.027 (-0.050, -0.004)	0.027	0.003 (-0.020, 0.025)	0.903	-0.008 (-0.030, 0.014)	0.988
cMFG	-0.021 (-0.044, 0.001)	0.080	-0.050 (-0.073, -0.028)	<0.001	0.013 (-0.009, 0.035)	0.793	0.002 (-0.020, 0.024)	0.988
FP	-0.001 (-0.024, 0.022)	0.939	-0.002 (-0.025, 0.020)	0.850	0.018 (-0.004, 0.040)	0.668	0.013 (-0.009, 0.034)	0.988
lOFC	-0.059 (-0.080, -0.037)	<0.001	-0.064 (-0.085, -0.043)	<0.001	-0.001 (-0.022, 0.019)	0.964	0.007 (-0.014, 0.027)	0.988
mOFC	-0.064 (-0.085, -0.043)	<0.001	-0.072 (-0.093, -0.051)	<0.001	-0.006 (-0.026, 0.014)	0.804	-0.009 (-0.029, 0.011)	0.988
PCL	-0.047 (-0.070, -0.025)	<0.001	-0.047 (-0.070, -0.025)	<0.001	0.009 (-0.013, 0.031)	0.804	0.003 (-0.019, 0.024)	0.988
OP	-0.041 (-0.064, -0.018)	0.001	-0.035 (-0.057, -0.013)	0.003	-0.008 (-0.030, 0.013)	0.804	-0.004 (-0.025, 0.018)	0.988
ORB	-0.039 (-0.061, -0.017)	0.001	-0.042 (-0.064, -0.020)	<0.001	0.007 (-0.014, 0.028)	0.804	0.018 (-0.003, 0.039)	0.832
TRI	-0.050 (-0.072, -0.027)	<0.001	-0.030 (-0.051, -0.008)	0.011	-0.007 (-0.028, 0.015)	0.804	0.001 (-0.020, 0.022)	0.988
PCG	-0.049 (-0.071, -0.028)	<0.001	-0.051 (-0.073, -0.030)	<0.001	-0.003 (-0.024, 0.018)	0.889	0.005 (-0.016, 0.026)	0.988
rACC	-0.021 (-0.044, 0.001)	0.077	-0.049 (-0.071, -0.027)	<0.001	0.004 (-0.018, 0.025)	0.881	-0.018 (-0.039, 0.003)	0.832
RMFG	-0.024 (-0.045, -0.003)	0.039	-0.047 (-0.068, -0.027)	<0.001	0.008 (-0.012, 0.028)	0.804	0.001 (-0.019, 0.021)	0.988
SFG	-0.033 (-0.054, -0.011)	0.005	-0.051 (-0.072, -0.031)	<0.001	-0.003 (-0.023, 0.018)	0.903	-0.005 (-0.025, 0.016)	0.988
**Parietal lobe**								
Bankssts	-0.025 (-0.048, -0.002)	0.045	-0.051 (-0.073, -0.029)	<0.001	-0.012 (-0.033, 0.010)	0.793	-0.004 (-0.026, 0.017)	0.988
EC	-0.025 (-0.048, -0.002)	0.046	-0.028 (-0.051, -0.006)	0.018	0.000 (-0.022, 0.022)	0.980	0.000 (-0.022, 0.021)	0.988
FG	-0.032 (-0.053, -0.010)	0.007	-0.050 (-0.071, -0.029)	<0.001	-0.013 (-0.033, 0.007)	0.793	-0.003 (-0.024, 0.017)	0.988
ITG	-0.055 (-0.076, -0.034)	<0.001	-0.063 (-0.083, -0.042)	<0.001	-0.001 (-0.021, 0.020)	0.964	0.000 (-0.020, 0.020)	0.988
MTG	-0.060 (-0.081, -0.038)	<0.001	-0.055 (-0.076, -0.034)	<0.001	-0.006 (-0.026, 0.015)	0.804	0.000 (-0.020, 0.020)	0.988
PHG	-0.030 (-0.053, -0.006)	0.021	-0.029 (-0.052, -0.006)	0.018	0.016 (-0.006, 0.039)	0.793	0.005 (-0.017, 0.027)	0.988
STG	-0.054 (-0.075, -0.032)	<0.001	-0.066 (-0.087, -0.045)	<0.001	-0.010 (-0.031, 0.011)	0.804	-0.008 (-0.028, 0.012)	0.988
TTG	-0.033 (-0.056, -0.010)	0.009	-0.041 (-0.064, -0.018)	0.001	0.008 (-0.014, 0.030)	0.804	-0.021 (-0.043, 0.000)	0.832
**Parietal lobe**								
IPL	-0.047 (-0.069, -0.025)	<0.001	-0.072 (-0.094, -0.051)	<0.001	-0.004 (-0.025, 0.017)	0.881	0.001 (-0.020, 0.021)	0.988
isthmus cingulate	-0.003 (-0.025, 0.018)	0.806	-0.042 (-0.064, -0.021)	<0.001	-0.012 (-0.033, 0.009)	0.793	-0.016 (-0.036, 0.005)	0.876
PoCG	-0.027 (-0.050, -0.005)	0.026	-0.044 (-0.066, -0.022)	<0.001	0.007 (-0.014, 0.028)	0.804	-0.007 (-0.028, 0.014)	0.988
PCC	-0.020 (-0.042, 0.002)	0.094	-0.043 (-0.065, -0.021)	<0.001	0.004 (-0.017, 0.025)	0.881	-0.020 (-0.041, 0.001)	0.832
PCUN	-0.039 (-0.061, -0.018)	0.001	-0.054 (-0.075, -0.033)	<0.001	0.006 (-0.014, 0.027)	0.804	0.006 (-0.015, 0.026)	0.988
SPL	-0.042 (-0.065, -0.020)	<0.001	-0.038 (-0.060, -0.016)	0.001	0.009 (-0.012, 0.031)	0.804	0.008 (-0.013, 0.029)	0.988
SMG	-0.047 (-0.069, -0.026)	<0.001	-0.041 (-0.062, -0.020)	<0.001	-0.005 (-0.026, 0.016)	0.843	0.001 (-0.020, 0.021)	0.988

**Occipital lobe**								
CUN	-0.017 (-0.039, 0.005)	0.157	-0.018 (-0.040, 0.004)	0.128	-0.011 (-0.032, 0.011)	0.804	-0.007 (-0.028, 0.014)	0.988
LOC	-0.020 (-0.041, 0.001)	0.079	-0.038 (-0.059, -0.018)	<0.001	-0.021 (-0.041, -0.001)	0.540	0.009 (-0.010, 0.029)	0.988
LG	-0.009 (-0.032, 0.014)	0.506	-0.029 (-0.052, -0.007)	0.014	-0.021 (-0.043, 0.001)	0.594	0.001 (-0.020, 0.023)	0.988
PCAL	-0.009 (-0.032, 0.015)	0.506	-0.006 (-0.029, 0.017)	0.628	-0.014 (-0.036, 0.008)	0.793	-0.012 (-0.034, 0.009)	0.988
**Insula lobe**								
INS	-0.055 (-0.076, -0.034)	<0.001	-0.078 (-0.098, -0.057)	<0.001	-0.011 (-0.031, 0.009)	0.793	-0.007 (-0.027, 0.013)	0.988

Abbreviations: IQR, interquartile range; FDR, false discovery rate; CRP, C-reactive protein; WBC, white blood cell; PLT, platelet counts; NLR, neutrophil-to-lymphocyte ratio.

a The abbreviations of the phenotypes were shown in Table S2.

b The '*P* value FDR' is a measure of the error detection rate obtained by correcting for the difference significance *P* value. A low '*P* value FDR value' (typically less than 0.05) suggests statistical significance.

Table S9. Associations of CRP and WBC with brain imaging phenotypes by age

Brain structure[a]	CRP			WBC		
	<65	≥65	*P* value FDR[b]	<65	≥65	*P* value FDR[b]
	β per IQR[b]	*β* per IQR[b]		*β* per IQR[b]	*β* per IQR[b]	
**Volume of subcortical structure**						
NAc	-0.063 (-0.087, -0.039)	0.015 (-0.052, 0.082)	0.031	-0.029 (-0.053, -0.006)	-0.012 (-0.079, 0.056)	0.634
AMYG	0.001 (-0.023, 0.025)	-0.034 (-0.102, 0.033)	0.331	-0.011 (-0.035, 0.012)	-0.013 (-0.081, 0.054)	0.957
CN	-0.057 (-0.081, -0.033)	-0.002 (-0.069, 0.065)	0.130	-0.066 (-0.090, -0.043)	0.002 (-0.065, 0.069)	0.059
HIP	-0.052 (-0.076, -0.028)	0.011 (-0.056, 0.078)	0.083	-0.023 (-0.047, 0.000)	0.045 (-0.022, 0.113)	0.060
GP	-0.086 (-0.109, -0.062)	-0.086 (-0.151, -0.020)	0.998	-0.056 (-0.079, -0.033)	-0.013 (-0.079, 0.054)	0.227
PUT	-0.057 (-0.079, -0.034)	-0.009 (-0.071, 0.053)	0.158	-0.063 (-0.085, -0.041)	0.007 (-0.056, 0.070)	0.038
THA	-0.098 (-0.121, -0.076)	0.007 (-0.056, 0.070)	0.002	-0.051 (-0.073, -0.029)	-0.021 (-0.085, 0.042)	0.378
**Volume of cortical structure**						
**Frontal lobe**						
cACC	-0.032 (-0.056, -0.008)	0.034 (-0.033, 0.101)	0.068	-0.054 (-0.078, -0.031)	-0.022 (-0.090, 0.046)	0.375
cMFG	-0.022 (-0.046, 0.003)	-0.052 (-0.121, 0.016)	0.409	-0.031 (-0.055, -0.007)	-0.003 (-0.072, 0.066)	0.439
FP	-0.033 (-0.056, -0.011)	-0.018 (-0.081, 0.045)	0.658	-0.052 (-0.074, -0.030)	-0.037 (-0.101, 0.026)	0.669
lOFC	-0.054 (-0.076, -0.032)	-0.062 (-0.124, 0.001)	0.819	-0.063 (-0.085, -0.041)	-0.060 (-0.123, 0.003)	0.921
mOFC	-0.062 (-0.085, -0.040)	-0.036 (-0.099, 0.026)	0.438	-0.055 (-0.077, -0.033)	-0.055 (-0.118, 0.008)	0.998
PCL	-0.027 (-0.052, -0.002)	-0.049 (-0.118, 0.021)	0.571	-0.037 (-0.061, -0.012)	0.039 (-0.031, 0.109)	0.046
OP	-0.061 (-0.084, -0.038)	0.009 (-0.055, 0.074)	0.040	-0.067 (-0.089, -0.044)	-0.061 (-0.125, 0.004)	0.866
ORB	-0.041 (-0.065, -0.016)	0.030 (-0.038, 0.099)	0.054	-0.044 (-0.068, -0.020)	-0.020 (-0.089, 0.049)	0.528
TRI	-0.048 (-0.071, -0.025)	-0.038 (-0.103, 0.027)	0.776	-0.074 (-0.097, -0.052)	-0.054 (-0.119, 0.011)	0.565
PCG	-0.010 (-0.033, 0.013)	0.054 (-0.010, 0.118)	0.064	-0.039 (-0.062, -0.017)	-0.069 (-0.133, -0.004)	0.399
rACC	-0.034 (-0.058, -0.011)	0.030 (-0.036, 0.096)	0.071	-0.047 (-0.070, -0.024)	-0.023 (-0.090, 0.043)	0.510
RMFG	-0.022 (-0.045, 0.002)	-0.005 (-0.071, 0.060)	0.647	-0.044 (-0.067, -0.021)	-0.031 (-0.098, 0.035)	0.717
SFG	-0.043 (-0.066, -0.020)	-0.006 (-0.070, 0.058)	0.277	-0.051 (-0.074, -0.029)	-0.079 (-0.144, -0.015)	0.422
**Temporal lobe**						
Bankssts	-0.033 (-0.060, -0.006)	0.045 (-0.032, 0.123)	0.061	-0.045 (-0.071, -0.018)	-0.022 (-0.100, 0.056)	0.591
EC	-0.026 (-0.054, 0.001)	-0.060 (-0.139, 0.019)	0.421	-0.030 (-0.057, -0.003)	-0.040 (-0.119, 0.040)	0.825
FG	-0.035 (-0.061, -0.010)	0.003 (-0.070, 0.075)	0.334	-0.050 (-0.075, -0.025)	-0.032 (-0.106, 0.041)	0.648
ITG	-0.056 (-0.081, -0.030)	-0.050 (-0.123, 0.022)	0.891	-0.063 (-0.088, -0.038)	-0.056 (-0.129, 0.017)	0.857
MTG	-0.065 (-0.090, -0.040)	-0.043 (-0.115, 0.029)	0.570	-0.050 (-0.075, -0.025)	-0.032 (-0.104, 0.041)	0.638
PHG	-0.020 (-0.048, 0.008)	-0.027 (-0.107, 0.054)	0.881	-0.037 (-0.065, -0.010)	0.030 (-0.051, 0.110)	0.125

STG	-0.066 (-0.092, -0.040)	0.028 (-0.046, 0.102)	0.018	-0.065 (-0.091, -0.040)	-0.068 (-0.142, 0.007)	0.952
TTG	-0.048 (-0.076, -0.020)	0.048 (-0.031, 0.127)	0.024	-0.041 (-0.068, -0.014)	-0.022 (-0.102, 0.057)	0.664
**Parietal lobe**						
IPL	-0.018 (-0.042, 0.006)	-0.009 (-0.075, 0.058)	0.793	-0.019 (-0.042, 0.005)	-0.010 (-0.077, 0.057)	0.810
isthmus cingulate	-0.019 (-0.042, 0.003)	-0.025 (-0.087, 0.038)	0.878	-0.040 (-0.062, -0.018)	-0.022 (-0.085, 0.041)	0.592
PoCG	-0.009 (-0.034, 0.015)	-0.002 (-0.069, 0.066)	0.827	-0.029 (-0.052, -0.005)	-0.035 (-0.103, 0.033)	0.866
PCC	-0.010 (-0.034, 0.015)	0.000 (-0.068, 0.068)	0.793	-0.007 (-0.030, 0.017)	0.000 (-0.069, 0.069)	0.865
PCUN	-0.018 (-0.042, 0.006)	-0.009 (-0.075, 0.058)	0.793	-0.019 (-0.042, 0.005)	-0.010 (-0.077, 0.057)	0.810
SPL	-0.019 (-0.042, 0.003)	-0.025 (-0.087, 0.038)	0.878	-0.040 (-0.062, -0.018)	-0.022 (-0.085, 0.041)	0.592
SMG	-0.009 (-0.034, 0.015)	-0.002 (-0.069, 0.066)	0.827	-0.029 (-0.052, -0.005)	-0.035 (-0.103, 0.033)	0.866
**Occipital lobe**						
CUN	-0.025 (-0.052, 0.002)	0.007 (-0.070, 0.084)	0.441	-0.023 (-0.049, 0.003)	-0.037 (-0.115, 0.040)	0.733
LOC	-0.023 (-0.049, 0.002)	0.005 (-0.067, 0.076)	0.469	-0.037 (-0.061, -0.012)	-0.021 (-0.093, 0.052)	0.677
LG	-0.018 (-0.045, 0.010)	0.028 (-0.050, 0.106)	0.274	-0.036 (-0.062, -0.009)	-0.026 (-0.105, 0.052)	0.825
PCAL	-0.019 (-0.047, 0.008)	0.004 (-0.075, 0.083)	0.582	-0.016 (-0.043, 0.011)	-0.007 (-0.087, 0.073)	0.825
**Insula lobe**						
INS	-0.056 (-0.078, -0.034)	-0.046 (-0.107, 0.015)	0.765	-0.078 (-0.099, -0.056)	-0.078 (-0.140, -0.017)	0.980

Abbreviations: IQR, interquartile range; FDR, false discovery rate; CRP, C-reactive protein; WBC, white blood cell.

a The abbreviations of the phenotypes were shown in Table S2.

b The '*P* value FDR' is a measure of the error detection rate obtained by correcting for the difference significance *P* value. A low '*P* value FDR value' (typically less than 0.05) suggests statistical significance.

Table S10. Associations of PLT and NLR with brain imaging phenotypes by age

Brain structure[a]	PLT			NLR		
	<65	≥65	*P* value FDR[b]	<65	≥65	*P* value FDR[b]
	β per IQR[b]	*β* per IQR[b]		*β* per IQR[b]	*β* per IQR[b]	
**Volume of subcortical structure**						
NAc	-0.004 (-0.027, 0.018)	-0.026 (-0.096, 0.045)	0.573	0.013 (-0.009, 0.036)	-0.081 (-0.146, -0.015)	0.008
AMYG	-0.024 (-0.047, -0.001)	-0.043 (-0.114, 0.027)	0.612	0.009 (-0.014, 0.031)	-0.055 (-0.121, 0.011)	0.073
CN	-0.006 (-0.029, 0.017)	0.052 (-0.019, 0.122)	0.123	0.014 (-0.009, 0.036)	0.000 (-0.066, 0.065)	0.693
HIP	-0.016 (-0.039, 0.007)	0.023 (-0.047, 0.094)	0.298	-0.004 (-0.027, 0.018)	-0.096 (-0.161, -0.030)	0.010
GP	-0.026 (-0.048, -0.003)	-0.022 (-0.092, 0.047)	0.932	0.007 (-0.015, 0.029)	-0.043 (-0.108, 0.021)	0.147
PUT	-0.014 (-0.035, 0.007)	-0.015 (-0.081, 0.050)	0.970	0.020 (-0.001, 0.041)	-0.015 (-0.076, 0.046)	0.284
THA	-0.004 (-0.027, 0.018)	-0.026 (-0.096, 0.045)	0.573	0.013 (-0.009, 0.036)	-0.081 (-0.146, -0.015)	0.008
**Volume of cortical structure**						
**Frontal lobe**						
cACC	-0.019 (-0.040, 0.003)	-0.016 (-0.082, 0.051)	0.933	0.011 (-0.010, 0.032)	-0.061 (-0.122, 0.001)	0.031
cMFG	-0.002 (-0.026, 0.022)	0.053 (-0.021, 0.126)	0.164	-0.005 (-0.029, 0.018)	-0.031 (-0.099, 0.038)	0.495
FP	0.012 (-0.011, 0.035)	0.021 (-0.050, 0.093)	0.808	0.007 (-0.016, 0.030)	-0.039 (-0.106, 0.027)	0.195
lOFC	0.022 (-0.001, 0.045)	-0.016 (-0.087, 0.055)	0.317	0.018 (-0.004, 0.041)	-0.035 (-0.101, 0.031)	0.135
mOFC	-0.004 (-0.026, 0.017)	0.028 (-0.039, 0.095)	0.362	0.013 (-0.008, 0.035)	-0.050 (-0.112, 0.012)	0.061
PCL	-0.008 (-0.029, 0.013)	0.014 (-0.052, 0.080)	0.531	-0.003 (-0.025, 0.018)	-0.058 (-0.120, 0.003)	0.097
OP	0.008 (-0.015, 0.031)	0.018 (-0.053, 0.088)	0.799	0.004 (-0.019, 0.026)	-0.005 (-0.071, 0.061)	0.808
ORB	-0.005 (-0.028, 0.018)	-0.040 (-0.111, 0.031)	0.362	0.009 (-0.014, 0.031)	-0.111 (-0.177, -0.045)	0.001
TRI	0.008 (-0.015, 0.03)	0.002 (-0.067, 0.071)	0.884	0.032 (0.010, 0.054)	-0.097 (-0.161, -0.033)	<0.001
PCG	-0.006 (-0.029, 0.016)	-0.011 (-0.080, 0.059)	0.903	0.006 (-0.016, 0.028)	-0.038 (-0.103, 0.026)	0.205
rACC	-0.007 (-0.029, 0.015)	0.033 (-0.035, 0.101)	0.276	0.010 (-0.012, 0.032)	-0.037 (-0.100, 0.027)	0.174
RMFG	0.006 (-0.016, 0.029)	-0.023 (-0.092, 0.047)	0.428	-0.011 (-0.033, 0.011)	-0.074 (-0.138, -0.009)	0.072
SFG	0.011 (-0.010, 0.032)	-0.021 (-0.087, 0.045)	0.362	0.009 (-0.012, 0.030)	-0.066 (-0.127, -0.005)	0.022
**Temporal lobe**						
Bankssts	-0.013 (-0.036, 0.010)	0.005 (-0.066, 0.076)	0.629	0.001 (-0.022, 0.024)	-0.051 (-0.117, 0.015)	0.143
EC	-0.001 (-0.024, 0.023)	0.004 (-0.068, 0.076)	0.897	0.002 (-0.021, 0.025)	-0.021 (-0.088, 0.046)	0.523
FG	-0.015 (-0.037, 0.006)	0.009 (-0.057, 0.076)	0.491	-0.002 (-0.023, 0.020)	-0.019 (-0.081, 0.043)	0.606
ITG	-0.002 (-0.023, 0.020)	0.007 (-0.058, 0.073)	0.799	0.006 (-0.015, 0.027)	-0.047 (-0.108, 0.015)	0.113
MTG	-0.004 (-0.025, 0.017)	-0.024 (-0.090, 0.042)	0.574	0.007 (-0.014, 0.028)	-0.055 (-0.116, 0.006)	0.062
PHG	0.016 (-0.008, 0.040)	0.020 (-0.053, 0.094)	0.908	0.006 (-0.018, 0.029)	-0.003 (-0.071, 0.065)	0.809

STG	-0.010 (-0.031, 0.012)	-0.013 (-0.080, 0.055)	0.928	-0.005 (-0.026, 0.017)	-0.035 (-0.098, 0.028)	0.368
TTG	0.008 (-0.016, 0.031)	0.013 (-0.060, 0.085)	0.899	-0.017 (-0.040, 0.006)	-0.061 (-0.128, 0.007)	0.227
**Parietal lobe**						
IPL	-0.001 (-0.023, 0.021)	-0.033 (-0.101, 0.036)	0.390	0.007 (-0.015, 0.029)	-0.051 (-0.115, 0.012)	0.090
isthmus cingulate	-0.015 (-0.037, 0.007)	0.015 (-0.053, 0.083)	0.415	-0.009 (-0.031, 0.013)	-0.072 (-0.135, -0.009)	0.063
PoCG	0.004 (-0.019, 0.026)	0.041 (-0.029, 0.110)	0.320	-0.006 (-0.028, 0.016)	-0.012 (-0.077, 0.053)	0.867
PCC	0.001 (-0.021, 0.023)	0.034 (-0.036, 0.103)	0.381	-0.013 (-0.035, 0.009)	-0.083 (-0.147, -0.018)	0.045
PCUN	0.007 (-0.014, 0.029)	-0.004 (-0.072, 0.064)	0.752	0.009 (-0.013, 0.031)	-0.023 (-0.086, 0.040)	0.341
SPL	0.014 (-0.008, 0.037)	-0.042 (-0.112, 0.028)	0.135	0.015 (-0.008, 0.037)	-0.048 (-0.113, 0.018)	0.076
SMG	-0.007 (-0.029, 0.014)	0.017 (-0.050, 0.085)	0.493	0.005 (-0.016, 0.027)	-0.039 (-0.102, 0.024)	0.194
**Occipital lobe**						
CUN	-0.010 (-0.032, 0.013)	-0.017 (-0.087, 0.053)	0.850	-0.005 (-0.027, 0.017)	-0.025 (-0.090, 0.040)	0.559
LOC	-0.017 (-0.038, 0.004)	-0.059 (-0.124, 0.007)	0.238	0.017 (-0.004, 0.038)	-0.059 (-0.120, 0.002)	0.020
LG	-0.019 (-0.042, 0.004)	-0.042 (-0.113, 0.029)	0.548	0.004 (-0.018, 0.027)	-0.026 (-0.092, 0.040)	0.395
PCAL	-0.015 (-0.038, 0.009)	-0.008 (-0.08, 0.064)	0.870	-0.009 (-0.032, 0.014)	-0.038 (-0.105, 0.029)	0.425
**Insula lobe**						
INS	-0.015 (-0.036, 0.006)	0.027 (-0.037, 0.092)	0.222	-0.003 (-0.024, 0.018)	-0.041 (-0.102, 0.019)	0.235

Abbreviations: IQR, interquartile range; FDR, false discovery rate; PLT, platelet counts; NLR, neutrophil-to-lymphocyte ratio.

a The abbreviations of the phenotypes were shown in Table S2.

b The '*P* value FDR' is a measure of the error detection rate obtained by correcting for the difference significance *P* value. A low '*P* value FDR value' (typically less than 0.05) suggests statistical significance.

Table S11. Associations of CRP and WBC with brain imaging phenotypes by sex

Brain structure[a]	CRP			WBC		
	Male	Female	*P* value FDR[b]	Male	Female	*P* value FDR[b]
	β per IQR[b]	*β* per IQR[b]		*β* per IQR[b]	*β* per IQR[b]	
**Volume of subcortical structure**						
NAc	-0.053 (-0.085, -0.020)	-0.056 (-0.087, -0.025)	0.890	-0.080 (-0.112, -0.048)	0.021 (-0.010, 0.051)	<0.001
AMYG	0.009 (-0.023, 0.042)	-0.014 (-0.045, 0.017)	0.299	-0.005 (-0.037, 0.027)	-0.018 (-0.048, 0.013)	0.573
CN	-0.028 (-0.060, 0.005)	-0.072 (-0.103, -0.041)	0.050	-0.062 (-0.094, -0.030)	-0.056 (-0.087, -0.026)	0.794
HIP	-0.039 (-0.071, -0.007)	-0.050 (-0.081, -0.019)	0.627	-0.028 (-0.060, 0.004)	-0.005 (-0.036, 0.025)	0.313
GP	-0.078 (-0.109, -0.046)	-0.093 (-0.123, -0.063)	0.490	-0.072 (-0.103, -0.041)	-0.032 (-0.062, -0.003)	0.071
PUT	-0.033 (-0.064, -0.003)	-0.068 (-0.097, -0.039)	0.103	-0.086 (-0.116, -0.056)	-0.028 (-0.057, 0.000)	0.006
THA	-0.079 (-0.110, -0.049)	-0.093 (-0.122, -0.064)	0.513	-0.076 (-0.106, -0.046)	-0.023 (-0.052, 0.006)	0.012
**Volume of cortical structure**						
**Frontal lobe**						
cACC	-0.006 (-0.040, 0.028)	-0.042 (-0.075, -0.010)	0.126	-0.045 (-0.079, -0.012)	-0.010 (-0.041, 0.022)	0.125
cMFG	-0.024 (-0.057, 0.009)	-0.019 (-0.050, 0.012)	0.832	-0.078 (-0.110, -0.045)	-0.025 (-0.056, 0.006)	0.020
FP	-0.007 (-0.040, 0.026)	0.005 (-0.027, 0.036)	0.617	-0.031 (-0.063, 0.001)	0.024 (-0.007, 0.055)	0.014
lOFC	-0.040 (-0.071, -0.010)	-0.075 (-0.105, -0.046)	0.102	-0.084 (-0.114, -0.054)	-0.046 (-0.075, -0.017)	0.073
mOFC	-0.046 (-0.077, -0.016)	-0.079 (-0.108, -0.051)	0.117	-0.102 (-0.131, -0.072)	-0.045 (-0.074, -0.017)	0.007
PCL	-0.028 (-0.060, 0.005)	-0.065 (-0.096, -0.034)	0.101	-0.070 (-0.102, -0.038)	-0.027 (-0.058, 0.003)	0.059
OP	-0.031 (-0.064, 0.001)	-0.050 (-0.081, -0.019)	0.415	-0.058 (-0.090, -0.026)	-0.014 (-0.044, 0.017)	0.047
ORB	-0.041 (-0.073, -0.010)	-0.038 (-0.068, -0.007)	0.865	-0.068 (-0.099, -0.037)	-0.018 (-0.048, 0.011)	0.022
TRI	-0.051 (-0.083, -0.019)	-0.048 (-0.079, -0.018)	0.909	-0.056 (-0.087, -0.025)	-0.006 (-0.035, 0.024)	0.022
PCG	-0.050 (-0.081, -0.019)	-0.049 (-0.079, -0.019)	0.961	-0.084 (-0.114, -0.053)	-0.022 (-0.051, 0.007)	0.004
rACC	0.008 (-0.024, 0.040)	-0.048 (-0.079, -0.018)	0.011	-0.069 (-0.100, -0.038)	-0.031 (-0.061, -0.001)	0.079
RMFG	-0.022 (-0.052, 0.008)	-0.026 (-0.054, 0.003)	0.854	-0.089 (-0.118, -0.059)	-0.010 (-0.038, 0.018)	<0.001
SFG	-0.040 (-0.071, -0.010)	-0.026 (-0.055, 0.003)	0.497	-0.090 (-0.120, -0.060)	-0.016 (-0.045, 0.013)	<0.001
**Temporal lobe**						
Bankssts	0.008 (-0.025, 0.040)	-0.055 (-0.086, -0.024)	0.006	-0.067 (-0.099, -0.035)	-0.036 (-0.067, -0.006)	0.175
EC	-0.021 (-0.054, 0.012)	-0.029 (-0.061, 0.003)	0.718	-0.047 (-0.080, -0.015)	-0.011 (-0.042, 0.020)	0.111
FG	-0.009 (-0.039, 0.022)	-0.052 (-0.081, -0.023)	0.041	-0.065 (-0.094, -0.035)	-0.037 (-0.066, -0.009)	0.196
ITG	-0.050 (-0.080, -0.020)	-0.059 (-0.088, -0.030)	0.651	-0.076 (-0.106, -0.046)	-0.051 (-0.079, -0.022)	0.224
MTG	-0.047 (-0.077, -0.017)	-0.071 (-0.100, -0.042)	0.253	-0.074 (-0.103, -0.044)	-0.038 (-0.067, -0.010)	0.089
PHG	-0.018 (-0.052, 0.015)	-0.040 (-0.072, -0.008)	0.351	-0.045 (-0.078, -0.011)	-0.014 (-0.046, 0.017)	0.194

STG	-0.044 (-0.075, -0.013)	-0.063 (-0.092, -0.033)	0.376	-0.093 (-0.124, -0.063)	-0.041 (-0.070, -0.012)	0.015
TTG	-0.033 (-0.066, 0.001)	-0.033 (-0.065, -0.002)	0.971	-0.041 (-0.074, -0.009)	-0.041 (-0.072, -0.010)	0.992
IPL	-0.030 (-0.061, 0.001)	-0.062 (-0.092, -0.032)	0.141	-0.116 (-0.146, -0.085)	-0.033 (-0.062, -0.003)	<0.001
**Parietal lobe**						
isthmus cingulate	-0.004 (-0.036, 0.027)	-0.002 (-0.032, 0.027)	0.919	-0.055 (-0.085, -0.024)	-0.031 (-0.060, -0.002)	0.268
PoCG	-0.017 (-0.049, 0.015)	-0.037 (-0.067, -0.006)	0.360	-0.065 (-0.097, -0.034)	-0.025 (-0.055, 0.005)	0.069
PCC	-0.010 (-0.042, 0.022)	-0.029 (-0.059, 0.002)	0.402	-0.077 (-0.108, -0.045)	-0.012 (-0.042, 0.018)	0.003
PCUN	-0.026 (-0.057, 0.005)	-0.052 (-0.081, -0.022)	0.227	-0.086 (-0.117, -0.056)	-0.025 (-0.054, 0.004)	0.004
SPL	-0.032 (-0.064, 0.000)	-0.052 (-0.083, -0.021)	0.363	-0.058 (-0.089, -0.026)	-0.020 (-0.050, 0.010)	0.086
SMG	-0.042 (-0.073, -0.010)	-0.053 (-0.083, -0.023)	0.598	-0.053 (-0.084, -0.023)	-0.030 (-0.059, 0.000)	0.265
**Occipital lobe**						
CUN	-0.012 (-0.044, 0.020)	-0.022 (-0.052, 0.009)	0.659	-0.026 (-0.057, 0.006)	-0.010 (-0.040, 0.020)	0.476
LOC	-0.010 (-0.040, 0.020)	-0.029 (-0.058, 0.000)	0.365	-0.066 (-0.095, -0.036)	-0.013 (-0.041, 0.015)	0.011
LG	-0.010 (-0.043, 0.023)	-0.007 (-0.039, 0.024)	0.911	-0.044 (-0.076, -0.012)	-0.016 (-0.047, 0.015)	0.211
PCAL	0.001 (-0.032, 0.034)	-0.017 (-0.049, 0.014)	0.436	-0.004 (-0.036, 0.029)	-0.008 (-0.039, 0.023)	0.869
**Insula lobe**						
INS	-0.053 (-0.083, -0.024)	-0.056 (-0.084, -0.028)	0.893	-0.096 (-0.125, -0.066)	-0.061 (-0.089, -0.033)	0.093

Abbreviations: IQR, interquartile range; FDR, false discovery rate; CRP, C-reactive protein; WBC, white blood cell.

a The abbreviations of the phenotypes were shown in Table S2.

b The '*P* value FDR' is a measure of the error detection rate obtained by correcting for the difference significance *P* value. A low '*P* value FDR value' (typically less than 0.05) suggests statistical significance.

Table S12. Associations of PLT and NLR with brain imaging phenotypes by sex

Brain structure[a]	PLT			NLR		
	Male	Female	*P* value FDR[b]	Male	Female	*P* value FDR[b]
	β per IQR[b]	*β* per IQR[b]		*β* per IQR[b]	*β* per IQR[b]	
**Volume of subcortical structure**						
NAc	-0.012 (-0.045, 0.021)	-0.002 (-0.031, 0.027)	0.673	-0.082 (-0.112, -0.052)	0.089 (0.059, 0.119)	<0.001
AMYG	-0.037 (-0.071, -0.004)	-0.017 (-0.046, 0.012)	0.369	-0.003 (-0.034, 0.027)	0.007 (-0.023, 0.037)	0.633
CN	0.012 (-0.021, 0.045)	-0.011 (-0.040, 0.018)	0.298	-0.001 (-0.031, 0.029)	0.025 (-0.005, 0.055)	0.227
HIP	0.005 (-0.028, 0.038)	-0.025 (-0.054, 0.004)	0.178	-0.085 (-0.116, -0.055)	0.057 (0.027, 0.087)	<0.001
GP	-0.015 (-0.047, 0.018)	-0.033 (-0.062, -0.005)	0.392	-0.033 (-0.063, -0.004)	0.037 (0.007, 0.066)	0.001
PUT	-0.016 (-0.047, 0.015)	-0.013 (-0.040, 0.014)	0.897	-0.046 (-0.075, -0.018)	0.079 (0.051, 0.107)	<0.001
THA	0.004 (-0.027, 0.035)	-0.035 (-0.062, -0.008)	0.063	-0.076 (-0.104, -0.047)	0.082 (0.054, 0.110)	<0.001
**Volume of cortical structure**						
**Frontal lobe**						
cACC	0.019 (-0.015, 0.054)	-0.010 (-0.040, 0.020)	0.209	-0.039 (-0.070, -0.007)	0.023 (-0.009, 0.054)	0.007
cMFG	-0.003 (-0.036, 0.031)	0.025 (-0.004, 0.054)	0.218	-0.057 (-0.088, -0.027)	0.061 (0.031, 0.092)	<0.001
FP	0.032 (-0.002, 0.065)	0.008 (-0.021, 0.037)	0.297	-0.020 (-0.051, 0.010)	0.046 (0.015, 0.076)	0.003
lOFC	0.012 (-0.020, 0.043)	-0.011 (-0.038, 0.016)	0.285	-0.043 (-0.072, -0.014)	0.056 (0.027, 0.085)	<0.001
mOFC	0.013 (-0.018, 0.044)	-0.020 (-0.047, 0.007)	0.112	-0.057 (-0.085, -0.029)	0.038 (0.010, 0.066)	<0.001
PCL	0.011 (-0.022, 0.044)	0.007 (-0.022, 0.036)	0.851	-0.039 (-0.069, -0.008)	0.044 (0.014, 0.075)	<0.001
OP	-0.002 (-0.036, 0.031)	-0.013 (-0.042, 0.016)	0.639	-0.056 (-0.086, -0.026)	0.048 (0.018, 0.078)	<0.001
ORB	0.010 (-0.022, 0.042)	0.005 (-0.023, 0.033)	0.804	-0.045 (-0.074, -0.016)	0.081 (0.052, 0.110)	<0.001
TRI	-0.015 (-0.047, 0.018)	-0.001 (-0.029, 0.028)	0.520	-0.061 (-0.091, -0.031)	0.063 (0.033, 0.092)	<0.001
PCG	-0.003 (-0.035, 0.029)	-0.003 (-0.031, 0.024)	0.998	-0.043 (-0.072, -0.014)	0.053 (0.024, 0.082)	<0.001
rACC	-0.012 (-0.045, 0.020)	0.016 (-0.012, 0.045)	0.191	-0.057 (-0.087, -0.027)	0.021 (-0.008, 0.051)	<0.001
RMFG	0.020 (-0.011, 0.050)	-0.001 (-0.027, 0.026)	0.332	-0.045 (-0.073, -0.017)	0.047 (0.019, 0.075)	<0.001
SFG	-0.018 (-0.049, 0.013)	0.009 (-0.018, 0.036)	0.192	-0.066 (-0.094, -0.037)	0.056 (0.028, 0.085)	<0.001
**Temporal lobe**						
Bankssts	-0.001 (-0.034, 0.033)	-0.020 (-0.049, 0.009)	0.384	-0.042 (-0.072, -0.012)	0.033 (0.003, 0.064)	0.001
EC	0.001 (-0.032, 0.035)	-0.001 (-0.031, 0.028)	0.903	-0.038 (-0.069, -0.007)	0.037 (0.006, 0.068)	0.001
FG	0.000 (-0.031, 0.031)	-0.023 (-0.050, 0.004)	0.275	-0.051 (-0.080, -0.023)	0.044 (0.016, 0.073)	<0.001
ITG	0.012 (-0.019, 0.043)	-0.010 (-0.037, 0.016)	0.283	-0.045 (-0.073, -0.017)	0.046 (0.017, 0.074)	<0.001
MTG	0.014 (-0.017, 0.044)	-0.020 (-0.047, 0.006)	0.102	-0.042 (-0.071, -0.014)	0.042 (0.014, 0.071)	<0.001
PHG	0.025 (-0.010, 0.059)	0.010 (-0.020, 0.040)	0.528	-0.025 (-0.056, 0.007)	0.034 (0.003, 0.066)	0.009

STG	-0.004 (-0.036, 0.027)	-0.014 (-0.042, 0.013)	0.635	-0.056 (-0.085, -0.027)	0.040 (0.011, 0.069)	<0.001
TTG	-0.002 (-0.036, 0.032)	0.016 (-0.014, 0.045)	0.429	-0.040 (-0.071, -0.009)	-0.003 (-0.034, 0.028)	0.096
**Parietal lobe**						
IPL	0.012 (-0.020, 0.044)	-0.017 (-0.045, 0.011)	0.181	-0.060 (-0.089, -0.031)	0.061 (0.032, 0.090)	<0.001
isthmus cingulate	-0.010 (-0.041, 0.022)	-0.014 (-0.042, 0.014)	0.835	-0.026 (-0.055, 0.003)	-0.005 (-0.034, 0.024)	0.315
PoCG	0.018 (-0.014, 0.051)	-0.002 (-0.030, 0.027)	0.369	-0.049 (-0.079, -0.019)	0.035 (0.005, 0.065)	<0.001
PCC	0.014 (-0.018, 0.047)	-0.004 (-0.032, 0.025)	0.411	-0.054 (-0.084, -0.024)	0.013 (-0.016, 0.043)	0.002
PCUN	0.010 (-0.021, 0.042)	0.003 (-0.024, 0.031)	0.752	-0.040 (-0.069, -0.011)	0.051 (0.022, 0.080)	<0.001
SPL	0.023 (-0.010, 0.056)	-0.001 (-0.030, 0.027)	0.278	-0.032 (-0.062, -0.002)	0.049 (0.019, 0.079)	<0.001
SMG	0.007 (-0.025, 0.038)	-0.014 (-0.042, 0.013)	0.326	-0.033 (-0.062, -0.004)	0.034 (0.005, 0.063)	0.001
**Occipital lobe**						
CUN	-0.005 (-0.038, 0.027)	-0.015 (-0.043, 0.014)	0.680	-0.024 (-0.053, 0.006)	0.009 (-0.020, 0.039)	0.126
LOC	-0.023 (-0.054, 0.007)	-0.019 (-0.046, 0.007)	0.853	-0.018 (-0.046, 0.010)	0.037 (0.009, 0.065)	0.006
LG	-0.016 (-0.049, 0.017)	-0.025 (-0.054, 0.004)	0.680	-0.021 (-0.051, 0.010)	0.023 (-0.008, 0.053)	0.049
PCAL	0.000 (-0.034, 0.034)	-0.025 (-0.054, 0.005)	0.282	-0.007 (-0.038, 0.023)	-0.017 (-0.048, 0.014)	0.665
**Insula lobe**						
INS	-0.011 (-0.041, 0.019)	-0.011 (-0.037, 0.015)	0.996	-0.041 (-0.069, -0.014)	0.027 (-0.001, 0.055)	0.001

Abbreviations: IQR, interquartile range; FDR, false discovery rate; PLT, platelet counts; NLR, neutrophil-to-lymphocyte ratio.

a The abbreviations of the phenotypes were shown in Table S2.

b The '*P* value FDR' is a measure of the error detection rate obtained by correcting for the difference significance *P* value. A low '*P* value FDR value' (typically less than 0.05) suggests statistical significance.

Table S13. Sensitivity analysis of the main associations

Brain structures	Sensitivity analysis 1		Sensitivity analysis 2		Sensitivity analysis 3		Sensitivity analysis 4	
	***β* per IQR**	***P* value FDR**	***β* per IQR**	***P* value FDR**	***β* per IQR**	***P* value FDR**	***β* per IQR**	***P* value FDR**
**Volume of subcortical structure**								
NAc	-0.025 (-0.047, -0.003)	0.037	-0.025 (-0.047, -0.004)	0.032	-0.025 (-0.047, -0.004)	0.035	-0.019 (-0.041, 0.003)	0.130
AMYG	-0.014 (-0.036, 0.008)	0.235	-0.011 (-0.033, 0.010)	0.319	-0.012 (-0.034, 0.009)	0.279	-0.008 (-0.030, 0.014)	0.485
CN	-0.040 (-0.062, -0.018)	0.001	-0.039 (-0.060, -0.017)	0.001	-0.039 (-0.061, -0.018)	0.001	-0.036 (-0.058, -0.014)	0.004
HIP	-0.026 (-0.048, -0.004)	0.031	-0.027 (-0.049, -0.006)	0.022	-0.027 (-0.049, -0.005)	0.023	-0.023 (-0.045, 0.000)	0.075
GP	-0.062 (-0.084, -0.041)	<0.001	-0.062 (-0.083, -0.040)	<0.001	-0.062 (-0.084, -0.041)	<0.001	-0.059 (-0.081, -0.037)	<0.001
PUT	-0.039 (-0.059, -0.018)	0.001	-0.039 (-0.059, -0.019)	<0.001	-0.040 (-0.060, -0.020)	<0.001	-0.035 (-0.055, -0.014)	0.004
THA	-0.051 (-0.072, -0.031)	<0.001	-0.053 (-0.073, -0.033)	<0.001	-0.053 (-0.073, -0.033)	<0.001	-0.048 (-0.069, -0.027)	<0.001
**Volume of cortical structure**								
**Frontal lobe**								
cACC	-0.022 (-0.045, 0.001)	0.074	-0.020 (-0.042, 0.003)	0.115	-0.019 (-0.042, 0.004)	0.123	-0.017 (-0.040, 0.006)	0.169
cMFG	-0.022 (-0.044, 0.000)	0.069	-0.019 (-0.041, 0.003)	0.115	-0.019 (-0.041, 0.003)	0.112	-0.019 (-0.042, 0.003)	0.124
FP	0.001 (-0.021, 0.023)	0.921	0.006 (-0.015, 0.028)	0.570	0.006 (-0.015, 0.028)	0.559	0.006 (-0.016, 0.028)	0.589
lOFC	-0.054 (-0.074, -0.033)	<0.001	-0.047 (-0.068, -0.027)	<0.001	-0.048 (-0.068, -0.027)	<0.001	-0.049 (-0.069, -0.028)	<0.001
mOFC	-0.050 (-0.070, -0.030)	<0.001	-0.047 (-0.068, -0.027)	<0.001	-0.047 (-0.068, -0.027)	<0.001	-0.046 (-0.067, -0.026)	<0.001
PCL	-0.034 (-0.056, -0.012)	0.004	-0.031 (-0.052, -0.009)	0.011	-0.031 (-0.052, -0.009)	0.010	-0.032 (-0.055, -0.010)	0.010
OP	-0.034 (-0.056, -0.012)	0.004	-0.030 (-0.052, -0.009)	0.011	-0.032 (-0.053, -0.010)	0.008	-0.029 (-0.051, -0.007)	0.019
ORB	-0.024 (-0.045, -0.003)	0.039	-0.018 (-0.039, 0.003)	0.115	-0.019 (-0.040, 0.002)	0.110	-0.017 (-0.038, 0.005)	0.154
TRI	-0.033 (-0.054, -0.011)	0.005	-0.030 (-0.051, -0.009)	0.010	-0.031 (-0.053, -0.010)	0.008	-0.030 (-0.051, -0.008)	0.015
PCG	-0.046 (-0.068, -0.025)	<0.001	-0.044 (-0.064, -0.023)	<0.001	-0.044 (-0.065, -0.023)	<0.001	-0.042 (-0.064, -0.021)	0.001
rACC	-0.037 (-0.058, -0.015)	0.002	-0.031 (-0.052, -0.010)	0.010	-0.031 (-0.053, -0.010)	0.008	-0.026 (-0.047, -0.004)	0.037
RMFG	-0.019 (-0.039, 0.001)	0.079	-0.016 (-0.036, 0.004)	0.147	-0.016 (-0.036, 0.004)	0.144	-0.016 (-0.036, 0.005)	0.163
SFG	-0.039 (-0.059, -0.018)	0.001	-0.034 (-0.055, -0.014)	0.002	-0.035 (-0.055, -0.015)	0.002	-0.036 (-0.056, -0.015)	0.003
**Temporal lobe**								
Bankssts	-0.039 (-0.061, -0.017)	0.002	-0.039 (-0.061, -0.017)	0.001	-0.038 (-0.060, -0.017)	0.002	-0.037 (-0.059, -0.015)	0.004
EC	-0.015 (-0.038, 0.007)	0.200	-0.014 (-0.036, 0.008)	0.252	-0.014 (-0.036, 0.008)	0.239	-0.010 (-0.032, 0.013)	0.429
FG	-0.030 (-0.050, -0.009)	0.007	-0.026 (-0.046, -0.005)	0.022	-0.026 (-0.047, -0.006)	0.018	-0.023 (-0.044, -0.002)	0.053
ITG	-0.039 (-0.059, -0.018)	0.001	-0.035 (-0.055, -0.014)	0.002	-0.035 (-0.055, -0.015)	0.002	-0.033 (-0.054, -0.013)	0.004
MTG	-0.049 (-0.070, -0.029)	<0.001	-0.045 (-0.065, -0.025)	<0.001	-0.046 (-0.066, -0.025)	<0.001	-0.046 (-0.066, -0.025)	<0.001
PHG	-0.013 (-0.036, 0.010)	0.277	-0.012 (-0.035, 0.010)	0.306	-0.012 (-0.035, 0.010)	0.301	-0.013 (-0.036, 0.010)	0.294

STG	-0.049 (-0.070, -0.029)	<0.001	-0.048 (-0.069, -0.028)	<0.001	-0.049 (-0.070, -0.028)	<0.001	-0.047 (-0.068, -0.025)	<0.001
TTG	-0.040 (-0.062, -0.017)	0.001	-0.036 (-0.058, -0.014)	0.004	-0.037 (-0.059, -0.015)	0.003	-0.034 (-0.057, -0.011)	0.008
**Parietal lobe**								
IPL	-0.046 (-0.067, -0.024)	<0.001	-0.044 (-0.064, -0.023)	<0.001	-0.044 (-0.064, -0.023)	<0.001	-0.039 (-0.061, -0.018)	0.002
isthmus cingulate	-0.036 (-0.057, -0.015)	0.002	-0.030 (-0.050, -0.009)	0.010	-0.030 (-0.051, -0.010)	0.008	-0.029 (-0.050, -0.007)	0.017
PoCG	-0.034 (-0.055, -0.012)	0.004	-0.029 (-0.051, -0.008)	0.012	-0.029 (-0.051, -0.008)	0.012	-0.030 (-0.052, -0.009)	0.014
PCC	-0.023 (-0.045, -0.002)	0.045	-0.021 (-0.042, 0.001)	0.077	-0.021 (-0.042, 0.000)	0.075	-0.021 (-0.043, 0.000)	0.082
PCUN	-0.035 (-0.056, -0.014)	0.002	-0.030 (-0.051, -0.009)	0.010	-0.030 (-0.051, -0.009)	0.008	-0.028 (-0.050, -0.007)	0.017
SPL	-0.028 (-0.049, -0.006)	0.019	-0.021 (-0.042, 0.000)	0.077	-0.021 (-0.043, 0.000)	0.072	-0.022 (-0.044, 0.000)	0.077
SMG	-0.037 (-0.057, -0.016)	0.002	-0.034 (-0.055, -0.013)	0.003	-0.035 (-0.055, -0.014)	0.003	-0.035 (-0.056, -0.014)	0.004
**Occipital lobe**								
CUN	-0.015 (-0.037, 0.006)	0.183	-0.009 (-0.030, 0.013)	0.429	-0.009 (-0.031, 0.012)	0.392	-0.011 (-0.033, 0.011)	0.344
LOC	-0.030 (-0.050, -0.010)	0.006	-0.021 (-0.041, -0.001)	0.060	-0.022 (-0.042, -0.002)	0.049	-0.020 (-0.040, 0.001)	0.083
LG	-0.021 (-0.043, 0.001)	0.078	-0.016 (-0.037, 0.006)	0.189	-0.016 (-0.037, 0.006)	0.192	-0.018 (-0.040, 0.005)	0.154
PCAL	-0.015 (-0.037, 0.008)	0.211	-0.014 (-0.036, 0.008)	0.252	-0.014 (-0.036, 0.008)	0.247	-0.016 (-0.039, 0.006)	0.182
**Insula lobe**								
INS	-0.054 (-0.074, -0.034)	<0.001	-0.051 (-0.071, -0.032)	<0.001	-0.052 (-0.072, -0.033)	<0.001	-0.059 (-0.082, -0.037)	<0.001

i) The cohort was restricted to individuals with White European ancestry, as non-White participants only constitute approximately 3% of the dataset.

ii) INFLA-score were trimmed on minimum and maximum (-16 and 16) to alleviate the influence of the extreme values.

iii) Considering the effects of neuropsychiatric diseases on brain structure, we control the prevalence of dementia and mental health in analyses additionally.

iv) We further adjusted for telomere length in the model since it represents the degree of aging which could also be potential confounders in the associations.
